# Discovery of phosphotyrosine-binding oligopeptides with supramolecular target selectivity†

**DOI:** 10.1039/d1sc04420f

**Published:** 2021-12-07

**Authors:** Ana S. Pina, Leonor Morgado, Krystyna L. Duncan, Sara Carvalho, Henrique F. Carvalho, Arménio J. M. Barbosa, Beatriz de P. Mariz, Inês P. Moreira, Daniela Kalafatovic, Bruno M. Morais Faustino, Vishal Narang, Tong Wang, Charalampos G. Pappas, Isabel Ferreira, A. Cecília A. Roque, Rein V. Ulijn

**Affiliations:** Advanced Science Research Center (ASRC) at the Graduate Center, City University of New York (CUNY) NY 10031 USA ana.pina@fct.unl.pt; Associate Laboratory i4HB – Institute for Health and Bioeconomy, NOVA School of Science and Technology, NOVA University Lisbon 2829-516 Caparica Portugal cecilia.roque@fct.unl.pt; UCIBIO – Applied Molecular Biosciences Unit, Department of Chemistry, School of Science and Technology, NOVA University of Lisbon 2829-516 Caparica Portugal; Department of Pure & Applied Chemistry, University of Strathclyde 295 Cathedral Street Glasgow G1 1XL UK; CENIMAT/I3N, Department of Materials Science, School of Science and Technology, NOVA University of Lisbon 2829-516 Caparica Portugal; Imaging Facility of CUNY ASRC 85 St Nicholas Terrace New York 10031 USA; Hunter College of CUNY, Department of Chemistry and Biochemistry 695 Park Avenue New York 10065 USA; PhD Programs in Chemistry and Biochemistry, The Graduate Center of CUNY New York 10016 USA rulijn@gc.cuny.edu

## Abstract

We demonstrate phage-display screening on self-assembled ligands that enables the identification of oligopeptides that selectively bind dynamic supramolecular targets over their unassembled counterparts. The concept is demonstrated through panning of a phage-display oligopeptide library against supramolecular tyrosine-phosphate ligands using 9-fluorenylmethoxycarbonyl-phenylalanine-tyrosine-phosphate (Fmoc-F*p*Y) micellar aggregates as targets. The 14 selected peptides showed no sequence consensus but were enriched in cationic and proline residues. The lead peptide, KVYFSIPWRVPM-NH_2_ (P7) was found to bind to the Fmoc-F*p*Y ligand exclusively in its self-assembled state with *K*_D_ = 74 ± 3 μM. Circular dichroism, NMR and molecular dynamics simulations revealed that the peptide interacts with Fmoc-F*p*Y through the KVYF terminus and this binding event disrupts the assembled structure. In absence of the target micellar aggregate, P7 was further found to dynamically alternate between multiple conformations, with a preferred hairpin-like conformation that was shown to contribute to supramolecular ligand binding. Three identified phages presented appreciable binding, and two showed to catalyze the hydrolysis of a model *para*-nitro phenol phosphate substrate, with P7 demonstrating conformation-dependent activity with a modest *k*_cat_/*K*_M_ = 4 ± 0.3 × 10^−4^ M^−1^ s^−1^.

## Abstract 2

Phage-display screening on self-assembled tyrosine-phosphate ligands enables the identification of oligopeptides selective to dynamic supramolecular targets, with the lead peptide showing a preferred hairpin-like conformation and catalytic activity.

## Graphical abstract

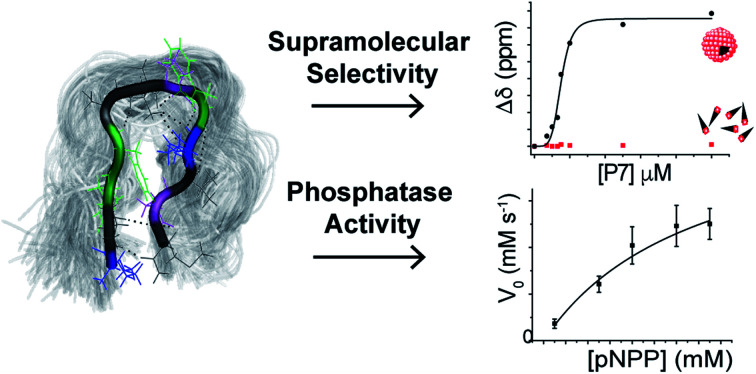

## Introduction

Oligopeptides are involved in many functions of biological relevance, including self-assembly, molecular recognition and catalysis, holding much promise as designed components for molecular biotechnology and biomimetic materials research.^1–6^ This notion has inspired efforts to design functional peptide modalities as functional (gene-encodable) tags for protein-based materials. Approaches for the discovery of short functional peptide modules include rational (computational) design,^7–9^ combinatorial screening methods,^10,11^ dynamic peptide libraries,^12^ phage display^13–16^ and hybrid computational-experimental methods^17,18^ that may be supported by machine learning algorithms.^19–21^ Phage display has successfully led to the identification of binding sequences towards protein surfaces,^22–24^ to a range of organic and inorganic nanostructures^13,15,16,25–29^ and small molecules bound to a surface, sometimes *via* flexible linkers.^14–16,30–32^ The identification of short peptides that select for self-assembled (amyloid) structures^33,34^ or precursors for catalytic self-assembly^15^ has clearly shown that short peptides can selectively bind stable supramolecular structures over unassembled counterparts.

In this work, we have developed methodology that brings together phage display with the field of designed supramolecular materials. We focus on an example from a popular class of supramolecular materials where aromatic groups are used to aid the self-assembly of biomolecules, here exemplified by 9-fluorenylmethoxycarbonyl-phenylalanine-tyrosine-phosphate (Fmoc-F*p*Y). Although there are affinity reagents (*e.g.*, antibodies/SH2 domains) that recognize phosphotyrosine moieties with high affinity and selectivity,^35–38^ the methodology proposed herein targets self-assembled structures that present the ligand of interest, the phosphotyrosine moiety, on a dynamic supramolecular surface. Although there is no known biological equivalent of this phosphorylated self-assembled material, the high-density presentation of functional groups is common in several supramolecular materials approaches. For our system, we show a first example where we target tyrosine-phosphate moieties exclusively in the self-assembled state.

The ability to identify peptide sequences that can bind to specific moieties presented by designed dynamic supramolecular structures can be useful in several contexts. For example, these peptide sequences can be incorporated into proteins that then bind to designed supramolecular structures; or identified peptide modalities may inform multi-component self-assembly strategies of relevance to dynamic structures that may incorporate significant disorder. For these, reversibility and dynamics can be important, so modest *K*_D_ values can be beneficial over the tight binding structures typically sought.

We also hypothesized that selection for supramolecular ligands may give rise to peptides that bind to ligands only in the supramolecular context; such an approach would be useful to target chemical functional groups to both biological and synthetic supramolecular structures.

**Fig. 1:**
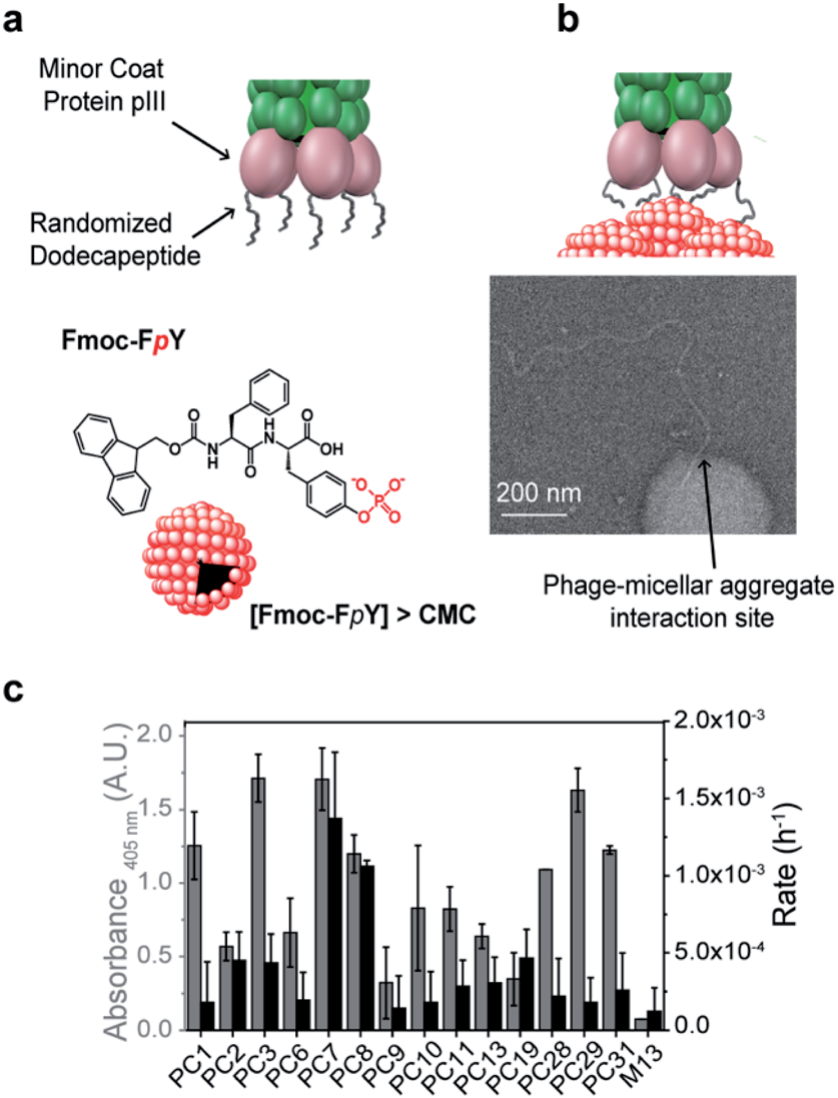
Phage display panning with supramolecular target selectivity. (a) Schematic representation of the M13 phage and phosphotyrosine supramolecular target, Fmoc-F*p*Y, (b) TEM imaging of the interaction of the tip of the phage of the lead phage clone PC7 towards Fmoc-F*p*Y (c) Phage clone date regarding binding assay by the colorimetric ELISA and phosphatase activity using the phosphatase model substrate *p*-nitrophenol phosphate (pNPP).

To demonstrate our concept, we chose Fmoc-F*p*Y as a simple target that was previously shown to form micellar aggregates^39^ through aggregation of the hydrophobic Fmoc-moieties and consequent presentation of phosphotyrosine on the surface. At 20 mM Fmoc-F*p*Y, forms aggregated micelles with a size distribution between 50-200 nm in diameter (Fig. 1 and S3†).

A commercially available dodecapeptide M13 phage display library was used to screen against self-assembled Fmoc-F*p*Y (Fig. 1a) and subsequently tested for binding this target and for the ability to hydrolyze the phosphate-ester bond (Fig. 1b and c). The selected phages were tested for binding and chemical reactivity, and the binding mode and efficiency of the top three lead sequences alongside appropriate controls were analyzed by spectroscopy techniques and molecular dynamics (MD) simulations. A number of strategic mutants of the lead P7 peptide sequence were also investigated to gain understanding of the mode of interaction.

## Results and discussion

Phage display peptide libraries were panned against Fmoc-F*p*Y above the critical micelle concentration (CMC) of 20 mM.^39^ During panning, the self-assembled Fmoc-F*p*Y target was free in solution, thus maximizing interactions between phages and targets, which contrasts previous biopanning strategies where self-assembling targets were typically pre-immobilized onto surfaces (*e.g*. polystyrene plates, glass substrates).^33,34^

A total of 33 colonies were obtained from the 3^rd^ round output (Table S1†), where an enrichment of binders was observed. The 33 phage colonies were subsequently sequenced and analyzed leading to 14 new peptide sequences (Table 1). While there was no sequence consensus in the hits obtained, sequences PC11 and PC13 were repeated four and two times, respectively. The chemical diversity obtained provides general features of binders.

**Table 1:** Phage clones sequences identified in the phage display panning

Phage clone	Sequence	# repetitions
PC1	STVRPLLMMDKY	1/33
PC2	HHLRIPYALDQT	1/33
PC3	DSAPSYNYRPSY	1/33
PC6	DYHDPSPPTLRK	1/33
PC7	KVYFSIPWRVPM	1/33
PC8	QVNGLGERSQQM	1/33
PC9	HSNDPRLITMRK	1/33
PC10	TCFAHTHNNFGH	1/33
PC11	DYHDPSLPTLRK	4/33
PC13	GNNPLHVHHDKR	2/33
PC19	RDYHPRDHTATW	1/33
PC28	IPGTAPPLARTG	1/33
PC29	KDFLPSPQTATW	1/33
PC31	VRAFSGEHSFVS	1/33

The phage panning led to the selection of 14 dodecapeptide sequences (Table 1, Fig. S1a†). The diversity of the amino acids incorporated at each of the 12 positions of these 14 sequences was first analyzed by the ratio between the frequency of each different amino acid at the *n* randomized position of the 12-mer sequence over the total 20 amino acids. This value was further normalized by the value 0.7 (ratio 14/20), corresponding to maximum of 14 different amino acids possible at *n* position (Fig. S1b†). Fig. S1b† shows that all positions of the 14 lead peptide sequences present an amino acid probability higher than 50%, except for position 5, which indicates a preferred type of amino acid at this position. The distribution of the amino acids observed at the 12 randomized positions for all the 14 peptide sequences was then analyzed and plotted in Fig. S1c.† Proline amino acid is statistically relevant specially between the positions at the middle of the sequence at positions 5 (43%), 7 (30%) and 9 (35%).

Although there is an overabundance of proline in the phage-displayed peptide population of the Ph.D-12™ libraries, this statistic overrepresentation is typically observed at positions 3 and 12,^40^ which contrasts with the results obtained here. As discussed later, the incorporation of a proline at the middle-sequence is important for peptide conformation,^5,41,42^ giving rise to β-turns, which was found to be important for the induced binding towards supramolecular phosphorylated-based aggregates.

All sequences (except for PC10) had at least one positively charged residue (R/K) or dyad RK/KR. Cationic amino acids are preferentially located at both termini as shown in Fig. S1c,† with a higher abundance at C-terminus, suggesting electrostatic interactions with the phosphate moiety. A distribution of negatively charged residues such as aspartic acid is also observed, near the N-terminus, which can counterbalance the presence of the positive charges in the peptide sequence. According to literature, the charged residues (R/K/D) are underrepresented in these type of phage-displayed peptides^40^ and these residues are typically associated with phosphate-binding.^43^

Another observation is the preferential presence of aromatic residues such as tyrosine and phenylalanine, at positions 3 and 4. This is interesting from the perspective of supramolecular aggregation interference, considering that the inner part of aggregates is enriched by aromatic fluorenyl, phenyl and phenol functional groups. Other amino acids with a frequency higher than 50% are found in a distributed manner throughout the peptide sequences, without position dependence or enrichment. These include histidine, hydrophobic residues such as leucine, alanine, valine, or polar residues such as threonine, glycine and serine. Overall, the selected peptides had several common compositional features with proline, aromaticity and charge playing a role in the selection.

The analysis of phage clones binding to supramolecular Fmoc-F*p*Y particles involved incubation of each amplified phage clone with Fmoc-F*p*Y at panning conditions, which was followed by buffer washing to avoid non-specific interactions and subsequent transfer of the phage/Fmoc-F*p*Y containing solutions to a 96-microplate for ELISA assay, to quantify the supramolecular target-bound phages (Fig. S2a†).

PC3, PC7 and PC29 were found to be the top binders towards Fmoc-F*p*Y (Fig. 1c), and interestingly, PC3 and PC7 present the highest combination of proline and aromatic residues with two and three respectively, followed by the PC29 with both two proline and aromatic residues. In contrast, the weakest binder, PC9, does not present any aromatic residue and only one proline residue. A common feature between all the peptide sequences is the presence of positively charged residues regardless of the extent of binding. In fact, PC9 is the lowest binder and the one containing the highest number of R/K residues which suggests non-specific binding towards Fmoc-F*p*Y.

Interactions of phages towards Fmoc-F*p*Y aggregates could also be imaged by TEM (Fig. 1b shows binding of the tip of amplified PC7 to the supramolecular target. Several dozen TEM images were obtained of samples of the PC7 and phage control M13 with Fmoc-F*p*Y). For PC7, we observed several examples of phage termini interacting with Fmoc-F*p*Y particles (additional examples are shown in Fig. S3†), while for the M13 phage none showed evidence of interaction.

Although it was not selected for in our screening setup, as hydrolysis of Fmoc-F*p*Y could not be detected, the phosphatase-like catalytic activity of the identified phage clones was also tested using *para*-nitrophenol phosphate (pNPP) with PC7 and PC8 observed to be significantly above background (Fig. 1c and S2b†).

Peptides P3, P7, and P29 were synthesized by solid-phase peptide synthesis and used in their free form in the following studies aiming to quantify binding and understand the interaction with the target. The P9 peptide sequence corresponding to the weakest binding phage was also studied as a negative control. The mode of binding of the free peptides P3, P7, P29 and P9 towards assembled Fmoc-F*p*Y was carried out in the same manner by using 1D^1^H Nuclear Magnetic Resonance (NMR). A solution of 20 mM Fmoc-F*p*Y was titrated with increasing concentrations of the respective peptide in the range 50–1000 μM and the chemical shift changes of the Fmoc-F*p*Y proton signals were monitored (as exemplified for P7 and Fmoc-F*p*Y in Fig. S4–S6†). The signals of the peptide could not be resolved because of the difference in concentrations between Fmoc-F*p*Y and peptides, as exemplified for P7 in Fig. S4.†

**Fig. 2:**
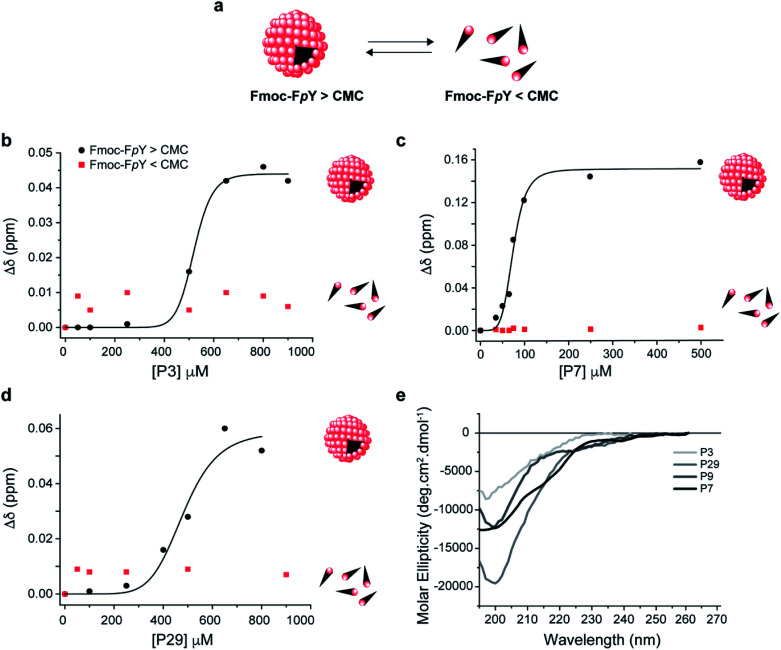
Supramolecular recognition of the top binding peptide (P3, P7 and P29) towards (a) Fmoc-F*p*Y and conformation analysis of the peptides. Binding curve for Fmoc-F*p*Y (1 mM, 20 mM) and (b) P3 (c) P7, and (d) P29. Interaction from 1D ^1^H NMR Chemical shift changes of the most downfield Fmoc-F*p*Y aromatic proton signal are shown. CD spectra of (e) P3, P7, P29 and P9 in 10 mM sodium phosphate pH 8.0. The fitting details can be found in the ESI.†

Analysis of the Fmoc-F*p*Y chemical shifts perturbation as a function of the peptide's concentration yielded binding curves that suggest a level of cooperativity in the interactions (Fig. 2 and S7†) for all the peptides except for P9 (Fig. S7†). The data were fitted to the Hill equation Δ*δ* = [peptide_free_]^*n*^/(*K*_D_^*n*^ + [peptide_free_]), where *K*_D_ is the dissociation constant and *n* the Hill coefficient that describes peptide cooperativity (Fig. 2). The *K*_D_ values that were obtained from the fitting of the peptide-induced chemical shift perturbation curves (Fig. 2a, S7† and Table 2) suggest the formation of a supramolecular-binding complex between Fmoc-F*p*Y and the peptides P3, P7 and P29 but with different *K*_D_ as shown in Table 2.

**Table 2:** The binding affinity for the various dodecapeptides for Fmoc-F*p*Y determined by NMR titration experiments

Peptide	Sequence	*K*_D_ (μM)	*r* ^2^
P3	DSAPSYNYRPSY-NH_2_	520 ± 7	0.99
P7	KVYFSIPWRVPM-NH_2_	74 ± 3	0.96
P29	KDFLPSPQTATW-NH_2_	488 ± 33	0.95
P9	HSNDPRLITMRK-NH_2_	n.d.	—

P7 was revealed to have the highest affinity towards Fmoc-F*p*Y with a *K*_D_ of 74 ± 3 μM, which is in the same order of magnitude compared to the values obtained for the peptides with affinity for previously reported self-assembling structures.^33,34^

The data obtained for P3 and P29 showed comparable cooperative binding profiles as for P7 but with *K*_D_ values that were an order magnitude higher than P7 (Fig. 2, S7† and Table 2), revealing lower affinity of the P3 and P29 for the assembled target. In contrast, the P9 concentration range used was not sufficient to reach saturation of the complex, and therefore a value of *K*_D_ was not possible to be determined (Fig. S8†). These results are in accordance with the panning results. Effectively, the peptide with the highest binding affinity towards the supramolecular Fmoc-F*p*Y aggregates was P7, which corresponds to the lead phage clone binder.

To test whether the peptides P3, P7 and P29 for Fmoc-F*p*Y differentiate between the self-assembled target and the free molecules, the titration was repeated at a concentration where Fmoc-F*p*Y is not assembled (1 mM) (Fig. 2 and S9†). At this concentration, no chemical shift perturbations were observed, confirming that P3, P7 and P29 displays supramolecular target selectivity (Fig. 2). The shape of the curves for all peptides also indicates a mechanism that is similar to positive allosteric cooperativity (*n* > 1) (Fig. 2 and S7†), suggesting that binding of one peptide molecule facilitates the binding of additional molecules. For the three peptides, the CH and aromatic protons of the Fmoc group (4.1 and 7.2–7.9 ppm, respectively) showed the highest chemical shift perturbation, followed by the Hα of the phenylalanine (3.5 ppm), and the Hδ of phosphorylated tyrosine (7 ppm) (Fig. S5 and S7†). These observations reveal that the Fmoc group experiences a more dramatic change in its chemical environment upon binding when compared with the more exposed aromatic protons of the tyrosine (Fig. S7†), suggesting that peptide binding interferes with the Fmoc stacking within the micellar aggregates. This structural disruption is expected to play a role in the observed allosteric cooperativity.

To understand the impact of the peptides' conformation on the mode of interaction, circular dichroism (CD) studies were performed for all the peptide sequences (Fig. 2d). The CD spectra revealed that all peptides appear to adopt a random coil conformation with a minimum around 200 nm, except for P7 that revealed a β-hairpin-like conformation,^44^ with a minimum at 196 nm, a small shoulder at 210 nm, and a weak positive band around 230 nm (Fig. 2d).

P7 peptide is the lead binder with a β-hairpin-like conformation, which can indicate that the binding interaction can benefit from a defined conformation.

Inspection of the P7 sequence KVYFSIPWRPM-NH_2_ reveals that the optimal combination between the proline positioning, aromaticity, and the N-terminal sequence (KVYF) that contains both cationic and aromatic residue can provide a complement of the Fmoc-F*p*Y structure. Therefore, the respective mode of interaction of P7 with Fmoc-F*p*Y was then studied in more detail.

To understand the importance of the phosphate-moiety in mediating the binding mechanism towards Fmoc-F*p*Y, a control experiment was performed to assess binding between P7 and Fmoc-FY. Considering the propensity of Fmoc-FY to undergo gelation,^39^ the samples of Fmoc-FY were prepared at lower concentrations, specifically at below and above the critical assembly concentration. Concentrations were chosen according to the emission spectra of Fmoc-FY where fibers form without gelation (1 mM), and in absence of the fibers formed (0.1 mM) (Fig. S10a†). Fiber formation was also confirmed by TEM in Fig. S10.† Considering these results, the study of the binding of P7 towards Fmoc-FY was conducted by NMR below (0.1 mM) and above (1 mM) the critical assembly concentration. 1D ^1^H NMR chemical shift changes of the most downfield Fmoc-FYp aromatic proton signal were monitored to understand the mode of interactions of the lead peptide P7 and Fmoc-FY (Fig. S11†). The results shown in Fig. S12† revealed that in the absence of the phosphate-moiety, the binding event between P7 and Fmoc-F*p*Y does not occur for both Fmoc-FY concentrations tested, which implies a pivotal role for the phosphate-moiety at mediating the binding with P7, possibly through the positively charged residues R and K.

**Fig. 3:**
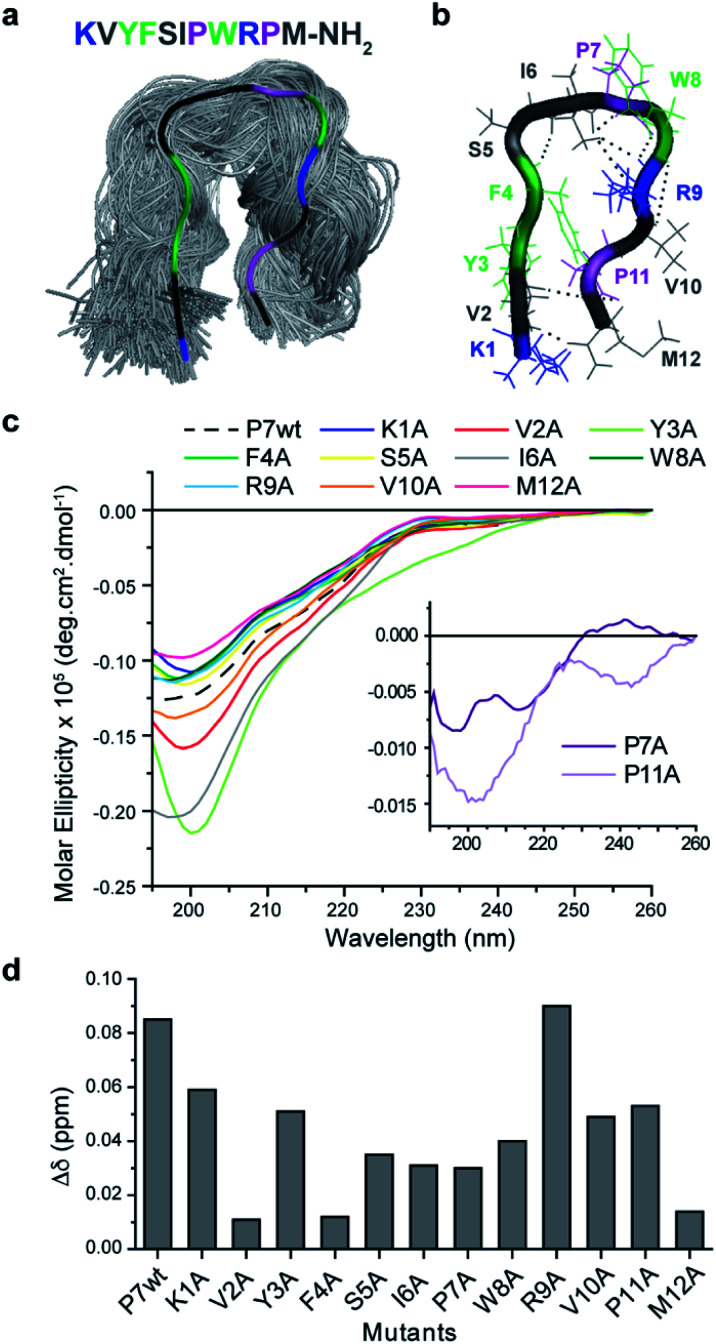
Conformational analysis of P7 and the respective alanine mutants (a) top cluster (30%) from MD simulations of P7 showing a β-hairpin-like structure maintained by specific (b) H-bonds. (c) CD spectra of P7 and Ala mutants in 10 mM sodium phosphate pH 8.0 and their (d) influence on the supramolecular binding complex of P7/Fmoc-F*p*Y evaluated by chemical shifts changes at the aromatic proton of the Fmoc group (7.9 ppm) of 20 mM Fmoc-F*p*Y.

The proposed hairpin-like P7 conformation in solution was also observed in MD simulations. As may be expected for a short peptide, P7 samples multiple conformations throughout the simulations (3 × 250 ns). However, for a significant (30%) part of the simulations the peptide adopts a hairpin-like conformation (Fig. 3a) which corroborates the CD spectra (Fig. 2b). This P7 conformation is stabilized by a cooperative exchange of backbone hydrogen bonds (Fig. 3b, S13 and S14†), and the key interactions are summarized in (Fig. S13 and S15†). Root mean square fluctuations (RMSF) of the C^α^ atoms from each residue shows that the core of the hairpin (residues 4–9, FSIPWR) is less dynamic compared to the termini (Fig. S16†).

Mutational analysis of the leading P7 peptide sequence (Fig. S15†) were then studied by 1D^1^H NMR to assess the contribution of each residue to the dynamic hairpin conformation of P7 (Fig. 3c) and to understand in more detail the mechanism of interaction of the supramolecular complex Fmoc-F*p*Y/P7 (Fig. 3d). Due to the precipitation of certain mutants at high concentrations (K1A, P7A, R9A and, P11A), this comparison was carried out at 75 μM where all mutants were soluble. Lower chemical shift differences are related to a decrease in the binding efficiency, and the results indicate that all residues contribute to binding, except for R9 (Fig. 3d). Overall, the KVYF terminus (in particular V and F) of the P7 has a stronger impact on binding compared to the C-terminal of P7, further confirming a role of aforementioned complementarity with Fmoc-F*p*Y. The core of the hairpin (4–8, FSIPW) is also critical for binding, thought to provide structural stability to the hairpin.

According to the CD spectra shown in Fig. 3c, the mutations that mostly affect P7 conformation are V2A, Y3A, I6A, P7A, and P11A, revealing their importance in maintaining the stability of the hairpin framework, V2, Y3, and I6 are involved in hydrogen bonding (Fig. S13 and S14†) and CH^α^ interactions (Fig. S15†) stabilizing the hairpin-like conformation. Moreover, these residues present lower RMSF values, indicating their contribution to lower flexibility of the hairpin conformation. Also, mutants P7A and P11A revealed a complete loss of hairpin-like structure, confirming the role of these Pro residues in stabilizing the conformation (Fig. 3c).

The NMR results, together with the evaluation of the mutants' conformation by CD, suggest that P7's hairpin-like conformation is essential for the formation of the supramolecular Fmoc-F*p*Y/P7 complex. We propose a mechanism involving selective binding towards the assembled state through interaction with the KVYF terminus which is facilitated by the hairpin-conformation and leads to partial disruption of the supramolecular aggregates. A similar induced-binding mechanism has been previously shown for particle-bound UTP-ligands^14^ and has been suggested for a heptapeptide bound to self-assembled peptide nanofibers.^34^

**Fig. 4:**
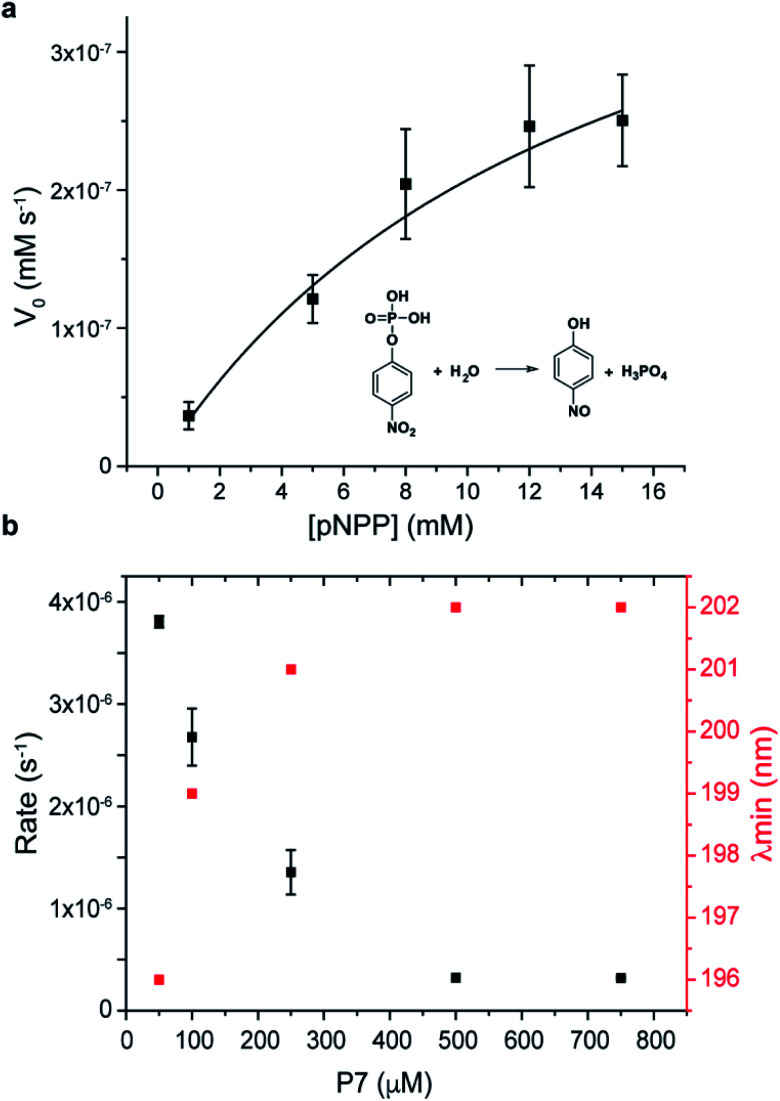
Kinetics of P7 peptide (a) towards the model phosphatase substrate pNPP. (a) Dependence of the initial rate of the reaction on pNPP substrate concentration. (b) Phosphatase activity of P7 was influenced by conformation as a function of P7 concentration. Error bars represent standard deviations of at least three independent measurements.

Finally, the catalytic activity that was observed during screening, and in the phage clone PC7 was investigated for the free oligopeptide P7 using the model substrate pNPP (Fig. 4a). The dependence of the initial rate of the reaction on substrate concentration follows Michaelis–Menten model, with *k*_cat_ = 10^−5^ s^−1^, *K*_M_ = 14 ± 4 mM, and catalytic efficiency of *k*_cat_/*K*_M_ = 4 ± 0.3 × 10^−4^ M^−1^ s^−1^. We note that the catalytic efficiency of P7 compares favourably to previously reported systems for phosphate ester hydrolysis, including a designed β-hairpin loop based on peptide-nucleic acid conjugate,^45^ a 42-residue helix-loop-helix peptide motif^46^ and peptide amphiphiles^47^ that showed catalytic activity when assembled into peptide nanofibers. A common feature between all is the presence of defined structural elements, reinforcing the importance of folding for chemical catalysis. An additional observation was that P7 concentration influences its catalytic rate, with a dramatic decrease in the catalytic rates observed as the concentration of P7 increases (Fig. 4b).

Coinciding with this reduction in catalytic activity is a conformational change of the peptide (Fig. S18†). The CD signature of the hairpin-like peptide changes as P7 concentration increases, with the minimum band blue-shifted to 202 nm and the weak positive band slightly red-shifted showing the maximum of the positive band at 230 nm and the disappearance of the shoulder at 210 nm (Fig. S16†). At concentrations higher than 100 μM, P7 revealed a CD signature of PP-II conformation, which is characterized by a negative band around 200–205 nm and by a weaker positive band around 225 nm (ref. 48 and 49) and expected to be enabled by the two proline residues. Also, at higher concentrations, P7 self-assembles into spherical aggregates, which were further confirmed using diffusion-ordered spectroscopy (DOSY) and imaged by TEM and AFM, shown in S19.†

This P7 self-assembly is related to a loss of β-hairpin-like structure and a conformational change of P7 to PP-II conformation, acting also as a conformation switch in the catalytic mechanism. The results suggest that the secondary framework of P7 of hairpin-like is the catalytic active conformation, as reported also in other peptide-based catalysts.^45,46,50^ We note that the hairpin to PP-II conformational switch observed at higher concentration was an unexpected result that was unrelated to the panning conditions, but an unintended consequence of the proline-rich sequence that was selected. The catalytic mechanism of P7 is currently not understood and will be the subject of future studies. We note that the catalytic activity for the supramolecular target Fmoc-F*p*Y (instead of the *para*-nitrophenol substrate) could not be conclusively demonstrated because the *k*_cat_ of P7 was not sufficient to hydrolyse Fmoc-F*p*Y.

While molecular recognition is the main driving during selection, the phosphatase activity was also used during the screening process. Although the catalytic activity was not sufficient to hydrolyse the Fmoc-F*p*Y phosphate ester bond, as initially intended and previously observed for an amide condensation target,^15^ the modest activity observed, and supramolecular regulation of this activity are interesting features to be exploited for further applications.

The peptide combines a remarkable combination of features in one sequence: binding to a supramolecular target, modest catalytic activity, and concentration-dependent supramolecular reconfiguration.

## Conclusions

A biopanning strategy based on supramolecular recognition led to the discovery of oligopeptides' sequences with chemical diversity and with sequence-dependent patterns. In particular, there is a clear preference of charged residues (R/K) located at sequence terminals, the presence of proline residues in the middle of the sequence, and aromatic residues (Y/F), preferentially positioned in the first half of the peptide. Although the chemical diversity was key to generate binders with different properties, it is envisaged that in future studies, a consensus sequence can be obtained by carrying out more rounds of biopanning.

The detailed binding mechanism of the lead oligopeptide-based sequences revealed micromolar range *K*_Ds_ (74–520 μM). This indicates reversible binding, and an ability to differentiate tyrosine phosphorylation between monomeric and aggregation states. The P3, P7 and P29 oligopeptides revealed selectivity for supramolecular over non-supramolecular states of phosphate molecules, enabling the creation of micromolar-affinity supramolecular complexes. The lead peptide P7 presented an optimal combination of chemical composition with a defined hairpin-like structure that allowed for an adaptive behavior towards supramolecular structures, *via* destabilization of the tyrosine-phosphorylated micellar aggregates. In future, we envision that the panning strategy developed in this work can also be applied to find binders to other dynamic targets such as the phase-separated biomolecular condensates.

Although there is no counterpart of the phosphorylated self-assembled Fmoc-F*p*Y in biology, high-density tyrosine phosphorylation is often seen in different diseases (*e.g.*, metabolic, neurodegenerative diseases). Therefore, the development of peptide modalities and functional materials that selectively target and perturb high-density phosphorylation can be incorporated in future diagnostic tools and therapeutics.

In addition, we also foresee that the chemical information dictating the ability of dodecapeptides at catalyzing phosphate ester hydrolysis phosphate expands the peptide field in aqueous media.

## Data availability

The data supporting the findings of this study are included in the main text and in the ESI file.†

## Author contributions

A. S. P., K. L. D, L. M., A. C. A. R., and R. V. U. conceived and designed the experiments. A. S. P., K. D. and S. C. carried out the phage display experiments. A. S. P, K. D., S. C. and B. P. M carried out the kinetic measurements and experimental conformational analysis. A. S. P. and L. M. conducted the binding NMR studies and DOSY NMR spectroscopy experiments. A. S. P, B. M. M. F., D. K. and V. N. performed the AFM imaging. I. F. and C. P. contributed for the self-assembly analysis. A. S. P., C. P. and T. W. performed the TEM experiments and contributed for sample preparation and data analysis. H. F. C., A. J. C and I. P. M. performed the computational studies. A. S. P, L. M., A. C. A. R. and R. V. U. co-wrote the paper.

## Conflicts of interest

There are no conflicts to declare.

## Supplementary Material

SC-013-D1SC04420F-s001
