# Supplementary material for: Discovery of phosphotyrosine-binding oligopeptides with supramolecular target selectivity
Source: Chem Sci. 2021 Dec 7;13(1):210–7. doi: 10.1039/d1sc04420f (PMC8694286; doi:10.1039/d1sc04420f)
Supplement: SC-013-D1SC04420F-s001 [file SC-013-D1SC04420F-s001.pdf]

Electronic Supplementary Material (ESI) for Chemical Science.
This journal is © The Royal Society of Chemistry 2021

Supplementary Information

Discovery of phosphotyrosine-binding oligopeptide with supramolecular target selectivity

Ana S. Pina [a,b,c,*], Leonor Morgado[b,c], Sara Carvalho[b,c], Krystyna L. Duncan[a,d], Henrique F. Carvalho[b,c], Arménio J. M. Barbosa[b,c], Beatriz de P. Mariz[b,c], Inês P. Moreira[b,c], Daniela Kalafatovic[a], Bruno M. Morais Faustino[e], Vishal Narang[a], Tong Wang[a,f] Charalampos G. Pappas[a,d], Isabel Ferreira[e], A. Cecília A. Roque[b,c, *] & Rein V. Ulijn[a,g,h,*]

a Dr. A.S. Pina, Dr. K.L. Duncan, Dr. D. Kalatatovic, Dr. V. Narang, Dr. T. Wang, Dr. C.G. Pappas and Prof. Dr. R.V. Ulijn
Advanced Science Research Center (ASRC) at the Graduate Center
City University of New York (CUNY)
NY 10031, USA
rulijn@gc.cuny.edu

b Dr. A.S. Pina, Dr. L. Morgado, S. Carvalho, Dr. H.F. Carvalho, Dr. A.J.M. Barbosa, B. P. Mariz, Dr. I. P.Moreira and Prof. Dr. A.C.A Roque
Associate Laboratory i4HB - Institute for Health and Bioeconomy,
NOVA School of Science and Technology, NOVA University Lisbon,
2829-516 Caparica, Portugal
ana.pina@fct.unl.pt; cecilia.roque@fct.unl.pt

c Dr. A.S. Pina, Dr. L. Morgado, S. Carvalho, Dr. H.F. Carvalho, Dr. A.J.M. Barbosa, B. P. Mariz, Dr. I. P.Moreira and Prof. Dr. A.C.A Roque
UCIBIO, Chemistry Department
School of Science and Technology, NOVA University of Lisbon
2829-516 Caparica, Portugal
ana.pina@fct.unl.pt; cecilia.roque@fct.unl.pt

d Dr. K.L. Duncan, Dr. C.G. Pappas and Prof. Dr. R.V. Ulijn
Department of Pure & Applied Chemistry
University of Strathclyde
295 Cathedral Street, Glasgow G1 1XL, UK

e Dr. B.M. M. Faustino and Prof. Dr. I. Ferreira
CENIMAT/I3N, Department of Materials Science
School of Science and Technology, NOVA University of Lisbon
2829-516 Caparica, Portugal

f Dr. T. Wang
Imaging Facility of CUNY ASRC,
85 St Nicholas Terrace, New York 10031, USA.

g Prof. Dr. R.V. Ulijn
Hunter College of CUNY, Department of Chemistry and Biochemistry
695 Park Avenue, New York, New York 10065, USA

h [7]PhD programs in Chemistry and Biochemistry
The Graduate Center of CUNY
New York 10016, USA.

MATERIALS AND METHODS

MATERIALS

Fmoc-F*p*Y with 95.0% purity was purchased from CSBio Co (CA, USA), the P7 peptide with 98.0% purity from China Peptides (China) and Caslo, the Ph.D.-12 phage display library, 2738 *E.coli* strain and M13 phage control from New England BioLabs (MA, USA), the primers from GenScript (NJ, USA) and the Chymotrypsin from Sigma Aldrich (NJ,USA).

METHODS

Phage Display

The Ph.D.-12 phage display peptide library kit (New England BioLabs) was used to select peptide binders with supramolecular recognition and phosphatase activity. For the bio-panning, the phage library (10^{11} pfu/mL) containing 10^9 phage different clones was mixed with 20 mM Fmoc-F*p*Y in 200 µL of 100 mM phosphate buffer pH 8.0 and incubated for 48h at room temperature. The phage particles were recovered by centrifugation (18000xg, 15 min), and then washed five times with Tris Buffered saline – 0.1% (v/v) Tween 20 (TBS-T) buffer. The Fmoc-peptide aggregates associated with the phages was removed by digestion with 200 µL of 10 mg mL-1 Chymotrypsin (Sigma-Aldrich) 30 min at 37ºC. The eluted phages were then amplified by infection into *E. coli* strain ER2738, followed by precipitation with 20% PEG-2.5M NaCl. The amplified and purified phages (input) and the eluted phages (output) were tittered on LB plates containing tetracycline, X-gal and IPTG. Three rounds of biopannig were performed. The phage supernatant products from the 3rd output plate was amplified and sequenced by GenScript.

Binding Assays by Colorimetric ELISA

The selected phage clones were amplified and then incubated with Fmoc-F*p*Y at the same conditions of biopanning. The last wash of Fmoc-phage aggregates was performed in coating buffer 100 mM bicarbonate/carbonate pH 8.6, immobilized onto a 96-well microplate (Maxisorb, Nunc) and then incubated overnight at 4ºC. After washing twice with TBS, 300 µL of Blocking Buffer (1% BSA in TBS-T 0.05% v/v) was added in each well and incubated 1h at room temperature. The respective wells were then washed with TBS-T (0.05% v/v) (5x) and incubated with 200 µL anti-M13 antibody monoclonal conjugated with HRP (1:2000) during 1h at room temperature. The wells were again washed with blocking buffer (4x) and once with TBS-T (0.05% v/v). The ABTS substrate (100 µL) was then added to each well and the absorbance at 405 nm was monitored in a SpectraMax i3 (Molecular Devices). Wild-type M13 phage has been used as a positive control of ELISA assay and as a negative control of Fmoc-F*p*Y binding. The experiments were performed in triplicate.

Kinetic Phosphatase Assays of the phage clones

The amplified phage clones and free peptide stock solutions (1mM and 0.5mM) were re-suspended in 100 mM phosphate buffer pH 8.0. The kinetic measurements were performed in a Jasco V-660 spectrophotometer monitoring absorbance of the product (*p*-nitrophenol) at 405 nm at 25ºC using a 1 cm quartz cuvette. p-Nitrophenol phosphate (pNPP) was used from a 100 mM stock solution to obtain a final concentration of 10 mM. A calibration curve was performed for different pNP concentrations ranging from 0 to 0.02 mM, for the quantification of pNP released. In case of kinetic assays with phage clones, a volume of 50 µL of phage solution previously amplified was added to 850 µL of PBS and 100 µL of pNPP stock solution. The initial velocity rate of pf each phage clone was obtained by using the data from the linear phase of pNP formed overtime (240h). The rate of catalysis was determined according to equation Rate (h^{-1}) = [Product formed] (mM) / ([P7] (mM) x time (h)). The experiments were performed in triplicate.

Phage Amplification

The phage plaques obtained from the 3rd round output plate were inoculated in 1-mL 2738 *E.coli* bacterial overnight culture diluted 1:100 and incubated at 37ºC at 220 rpm for 5h. The culture was then centrifuged at 12,000xg for 1 min. The supernatant was then removed to a fresh micro-centrifuge tube and re-centrifuged (12,000xg, 1min, 4°C). 80% of the supernatant was then transferred to a fresh tube and precipitated overnight with 150 µL of 20% PEG /2.5 M NaCl. The precipitated phage was then centrifuged at 12000xg and 4°C for 15 min and the supernatant was removed, and the pellet was re-centrifuged to remove residual supernatant. The pellet was re-suspended in 1 mL TBS. The solution was transferred to a new tube and spin at 12000xg, 4ºC for 5 min to pellet residual cells. The phage supernatant was precipitated with 200 µL of 20%PEG-2.5M NaCl and incubated on ice for 1h. The phage solution was centrifuged at 12000xg and 4°C for 15 min. The supernatant was again discarded, and the pellet was re-spin at 12000xg and 4°C for 5 min. The transparent pellet was re-suspended in 200 µL of 100 mM phosphate buffer pH 8.0.

TEM of Phage samples

TEM imaging was performed at the Advanced Science Research Center (ASRC), CUNY Imaging Facility using a FEI, TITAN Halo TEM operating at 300 Kv. Images were recorded in the low-dose mode (20 e − Å–2) on FEI CETA

16M camera (4,096 × 4,096 pixels). Carbon-coated grids were purchased from Electron Microscopy Sciences. The grids were firstly glow discharged in air for 30 s. 4.5 μL of sample was added to the grid and blotted down using filter paper after 60 s. The grids were then washed twice with distilled water and blotted with filter paper after 30 sec, to completely remove 100 mM sodium phosphate pH 8.0 buffer. For double negative staining, 4.5 μL of 2% aqueous uranyl acetate was applied twice (30 s each time) and the mixture blotted again using filter paper to remove excess. The dried grids coated with sample were then imaged.

NMR samples preparation and experiments

For NMR studies, a series of Fmoc-F*p*Y samples at 20mM, 1mM or Fmoc-FY with increasing amounts of p7 (35 μM, 50 μM, 65 μM, 75 μM, 100 μM, 250 μM and 500 μM) were prepared in 100 mM sodium phosphate buffer pH 8.0 in 92%H_2O/8%^2H_2O and incubated 48h at 25ºC. 1D-^{1}H NMR spectra were acquired on each sample with 1k scans at 25 °C. Samples with p7 mutants were prepared at different concentrations, and spectra acquired in the same conditions.

For the assignment of the Fmoc-F*p*Y signals the following spectra were acquired on a 20mM sample: 2D-^{1}H,^{13}C HSQC (Heteronuclear Single Quantum Coherence), 2D-^{1}H,^{1}H TOCSY (TOtal Correlation SpectroscopY) with 20 ms and 100 ms mixing-time and 2D-^{1}H,^{1}H ROESY (Rotating-frame Overhauser Effect SpectroscopY) with 250 ms mixing time.

The NMR experiments were acquired in a Bruker Avance III 600 spectrometer equipped with a triple-resonance cryoprobe (TCI). Spectra were processed using software TOPSPIN (Bruker Biospin, Karlsruhe, Germany) and peaks assigned with Sparky (TD Goddard and DG Kneller, Sparky 3, University of California, San Francisco, USA).

The dissociation constants K_D (μM) were determined in the OriginPro9 by fitting the data to the Hill Equation $\Delta\delta$= $[\text{Peptide}_{\text{free}}]^n / (K_D{}^n + [\text{Peptide}_{\text{free}}])$, where K_D is the dissociation constant and n the Hill coefficient that describes Peptide cooperativity. The datapoints used for each on the fitting is discriminated in the table below. The 1D ^{1}H NMR Chemical shift changes are of the most downfield Fmoc-F*p*Y aromatic proton signal.

Δδ (ppm)	[P3] (μM)	Δδ (ppm)	[P7] (μM)	Δδ (ppm)	[P29] (μM)
0	0	0	0	0	0
50	0	0.012	35	100	0.001
100	0	0.023	50	250	0.003
250	0,001	0.034	65	400	0.018
500	0.016	0.085	75	500	0.028
650	0.042	0.122	100	650	0.06
800	0.046	0.144	250	800	0.052
900	0.042	0.157	500		

Kinetics of P7 towards the model phosphatase substrate pNPP

The catalysis of free peptide was evaluated by using different peptide concentrations within the range 50 μM – 750 μM. In order to determine the kinetic parameters of free peptide, different substrate concentrations were tested with 50 μM of peptide. For this a volume of 50 μL from a peptide stock solution of 1mM was added to a 1 mL cuvette with 850 μL of 100 mM phosphate buffer pH 8.0 and 100 μL of substrate stock solution (10, 50, 80, 100, 120, 150) in 100 mM phosphate buffer pH 8.0. The initial velocity rate of each substrate concentration catalysed by the free peptide were obtained by using the data from the linear phase of pNP formed over time (240h). The kinetic parameters (V_{max} and K_M were determined by fitting the data to the Michaelis–Menten equation V_0= $V_{max}[S]/(K_M)$. k_{cat}/K_M values were obtained by fitting the linear regime of the Michaelis–Menten plot to $V_0 = (k_{cat}/K_M)$ [E][S]. The rate of catalysis was determined according to equation Rate (s^{-1}) = [Product formed] (mM) / ([P7] (mM) x time (s)). The experiments were performed in triplicates.

Diffusion ordered spectroscopy

Diffusion ordered spectroscopy (DOSY) spectra of P7 were acquired at 50 μM and 750 μM concentration using the pulse sequence from the Bruker library (ledbpgppr2s). The spectra were recorded with 2k scans for the lower concentration sample and 256 scans for the high concentration sample. The diffusion time was adjusted to 80 ms and the duration of the encoding/decoding gradient was calibrated to 4ms. The pulse gradient was increased from 2 to 95% of the maximum gradient strength using a linear ramp in 32 gradient steps. 32k data points in the F2 dimension were collected. DOSY experiments were acquired in a Bruker Avance III 400 spectrometer equipped with a triple resonance probe (TXI) and analyzed with TOPSPIN.

Circular Dichroism

The far-UV CD spectra (190-250 nm) was acquired with an Applied Photophysics Chirascan™ qCD spectrometer at 25ºC in a 1 × 1 mm square quartz cuvette under nitrogen flow, response of 3 s/nm and bandwidth of 1 nm. Each spectrum represents an average of three scans.

Computational Studies

Molecular Dynamics Simulations

MD simulations of p7 monomer peptide were performed in GROMACS 2016.5 simulation package[1] using the AMBER99SB-ILDN force field [2]. The Lys and Arg residues were modelled in the protonated state, with a positively charged N-terminal and an amidated C-terminal. Starting from the extended conformation, the peptide was placed in a cubic box of 197 nm^3. The box was then solvated with TIP3P[3] water molecules and 3 chloride counter-ions were added to neutralize the total charge with long-range electrostatics being treated with the Particle-Mesh Ewald algorithm[4]. The system was then energy-minimized in two steps to remove atom clashes and bond contacts: first by a steepest descent minimization algorithm (max 2000 steps), followed by a conjugated gradient algorithm (max 1000 steps). Equilibration of solvent molecules was made for 5 ns in a NPT ensemble, with the V-rescale thermostat at 300 K (time-constant of 1.6 ps), the isotropic Berendsen barostat at 1 bar (time-constant 5 ps) and with positional restrains for all peptide heavy-atoms with the LINCS algorithm with a force constant of 1000 kJ/mol/nm [2]. The integration step was 2 fs and coordinates were saved each 20 ps, Production runs in the NPT ensemble were made for 250 ns in 3 independent replicates in a total of 750 ns.

Cluster analysis of the 3 replicates was made using the method reported by Daura *et al.* [5]. The root means square RMSD matrix of 7501x7501 elements (equally spaced 100 ps frames) was calculated, with a minimum of 100 structures per cluster and a cut-off of 3 Å between backbone atoms. For each cluster, the centroid structure was obtained.

AFM Imaging

Samples were prepared by diluting 20 µl of the sample to a total volume of 100 µl of solution in deionized water. Then it was pipetted on a freshly cleaved mica sheet (G250–2 Mica sheets 1″ × 1 ″ × 0.006″ (Agar Scientific Ltd)) attached to an AFM support stub and left to air dry overnight in a dust-free environment, prior to imaging. For AFM measurements, samples of the assembled peptides were diluted 7.5-fold in deionized water in a total volume of 100. 15 µL of each of the diluted sample was deposited on top of a freshly cut mica substrate (G250–2 Mica sheets 1″ × 1 ″ × 0.006″, Agar Scientific Ltd) and let air dry overnight in a dust-free environment, prior to imaging. The images were obtained by scanning the mica surface in air under ambient conditions using a Multimode 8 and a FastScan Microscope (Bruker) operated in tapping mode. The AFM scans were taken at a resolution of 512 × 512 pixels. The images were analysed using NanoScope Analysis software Version 1.40.

Determination of the Critical Aggregation Concentration (CAC)

A stock solution of 2.5mM pyrene in methanol was prepared and then diluted 20-fold in methanol. 50 µl of the diluted pyrene solution was added to each of the different concentrations of peptide P7. The concentrations used were 0.05, 0.25, 0.75, 1.25, 2.5 and 5mM[6].These samples were excited at 334 nm in a Jasco FP-6500 spectrofluorometer. The ratio of the first (I) and third peak (III) λ_{max} values was plotted against peptide P7 concentration to determine the critical aggregation concentration [7].

RESULTS & DISCUSSION

Table S1: Phage titers (imput and output) with increasing rounds of selection.

# rounds	Colony-forming units (CFU)	
	imput	output
1	21 x 10^{10}	5 x 10^{3}
2	16 x 10^{6}	1 x 10^{2}
3	30 x 10^{7}	33 x10^{6}

a

#1 STVRPLLMMDKY
#2 HHLRIPYALDQT
#3 DSAPSYNYRPSY
#6 DYHDPSPPTLRK
#7 KVYFSIPWRVPM
#8 QVNGLGERSQQM
#9 HSNDPRLITMRK
#10 TCFAHTHNNFGH
#11 DYHDPSLPTLRK
#13 GNNPLHVHHDKR
#19 RDYHPRDHTATW
#28 IPGTAPPLARTG
#29 KDFLPSPQTATW
#31 VRAFSGEHSFVS

b

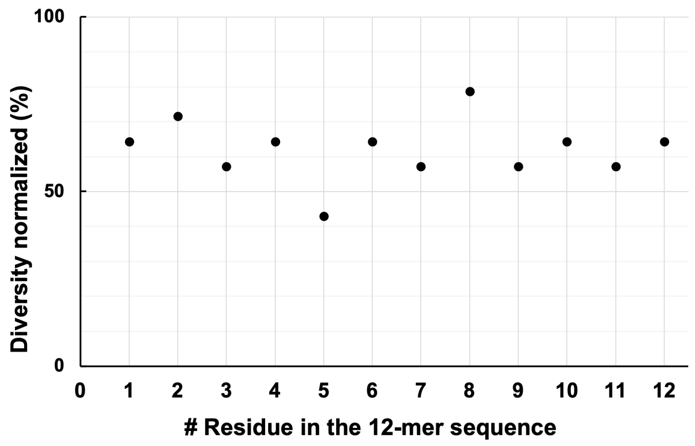

c

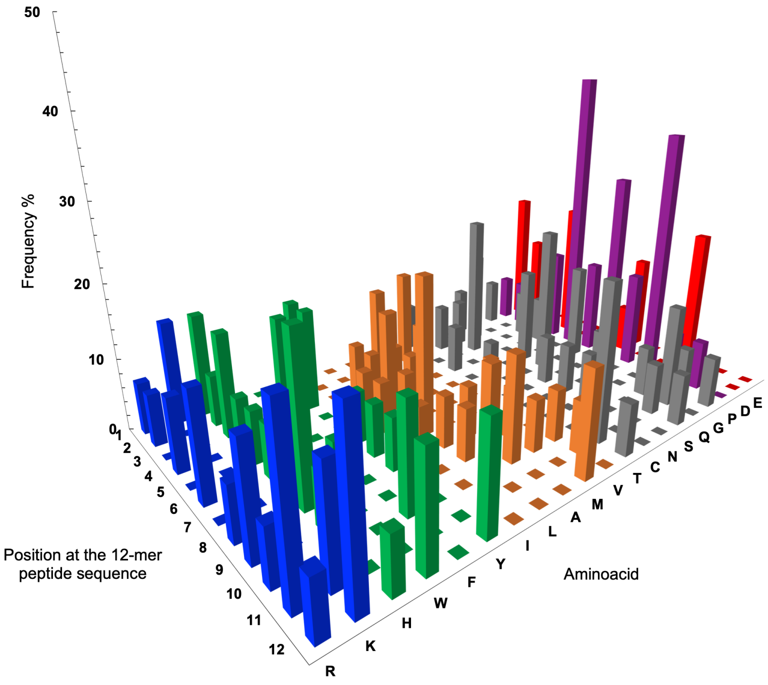

Supplementary Figure 1: Quantitative analysis of **(a)** phage clones obtained and the respective **(b)** diversity distribution of the aminoacids at the *n* randomized positions for the 14 12-mer peptide sequences. The diversity was normalized by a factor of 0.7 (ratio of 14/20), which corresponds to a maximum of 14 different aminoacids at the *n* position of the 14 peptide sequences, in a total of 20 aminoacids. **(c)** Frequency of the aminoacids incorporated at the n randomized positions of the 14 12-mer peptide sequences.

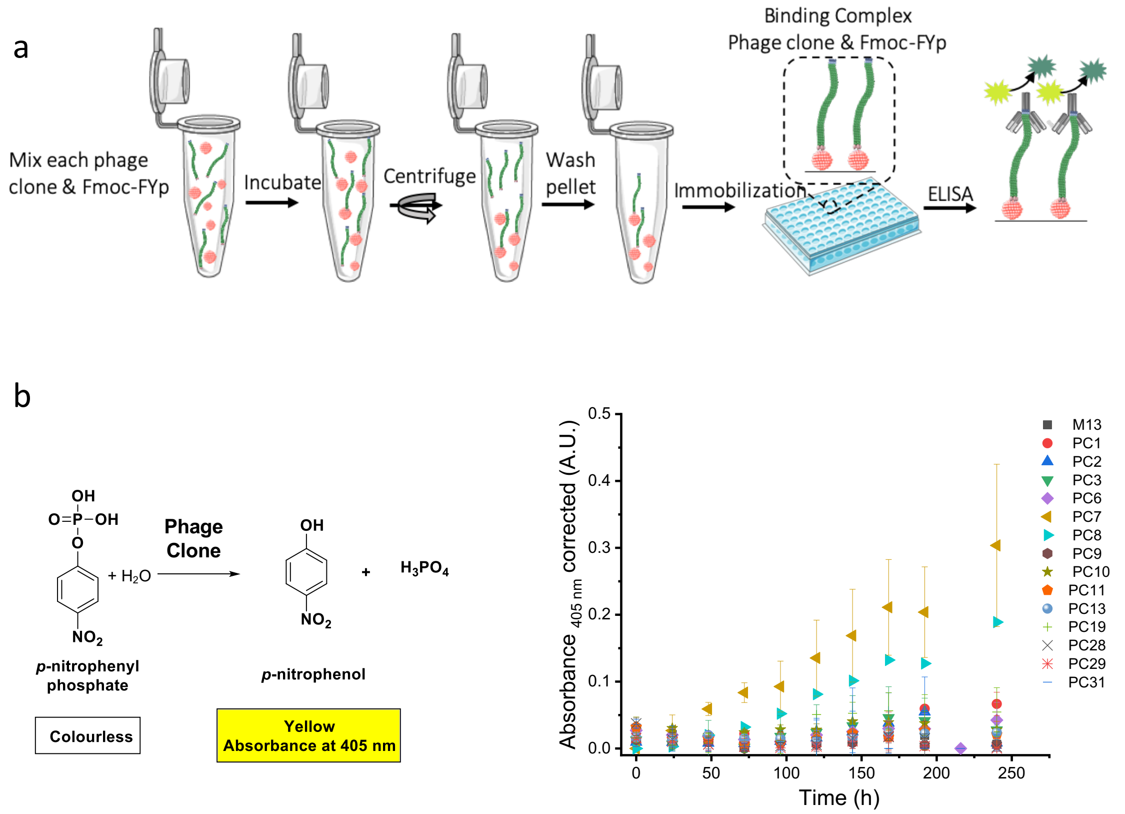

Supplementary Figure 2 – (a) Binding Assays by Colorimetric ELISA where the phage clones were incubated for 48h with Fmoc-F*p*Y micelles and then after buffer washing, the binding complex Fmoc-F*p*Y/phage clones (PC) were immobilized on a 96-well microplate by physical adsorption, followed by ELISA assay. (b) The Phosphatase Activity of the phage clones was carried out by using the phosphatase model substrate p-nitrophenol phosphate (pNPP). The hydrolysis of the pNPP by the 14 phage clones obtained and M13 phage was monitored by the absorbance at 405 nm from the chromogenic product p-nitrophenol.

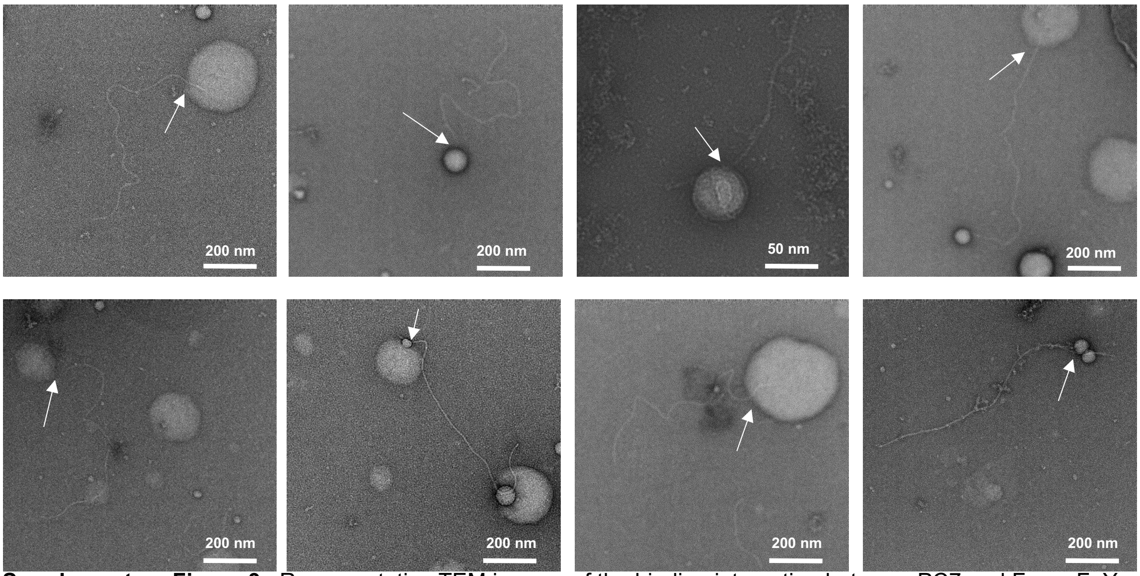

Supplementary Figure 3– Representative TEM images of the binding interaction between PC7 and Fmoc-F*p*Y.

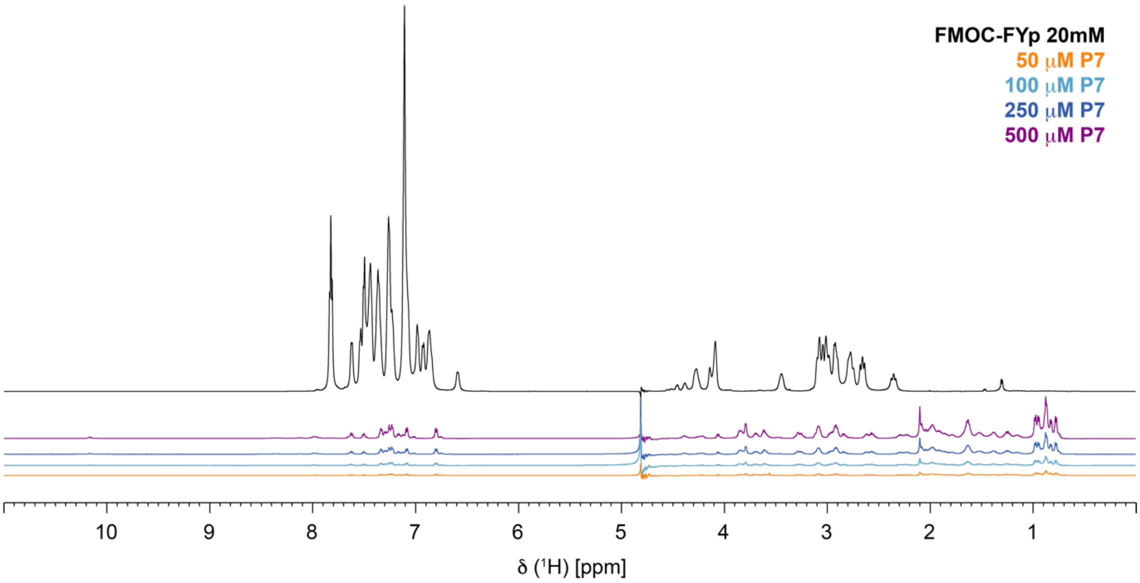

Supplementary Figure 4 Comparison of the 1D-^{1}H NMR spectra of 20 mM Fmoc-F*p*Y and of P7 at different concentrations of P7 (50, 100, 250 and 500 μM) in 100mM sodium phosphate pH 8 at 25 °C and 600 MHz.

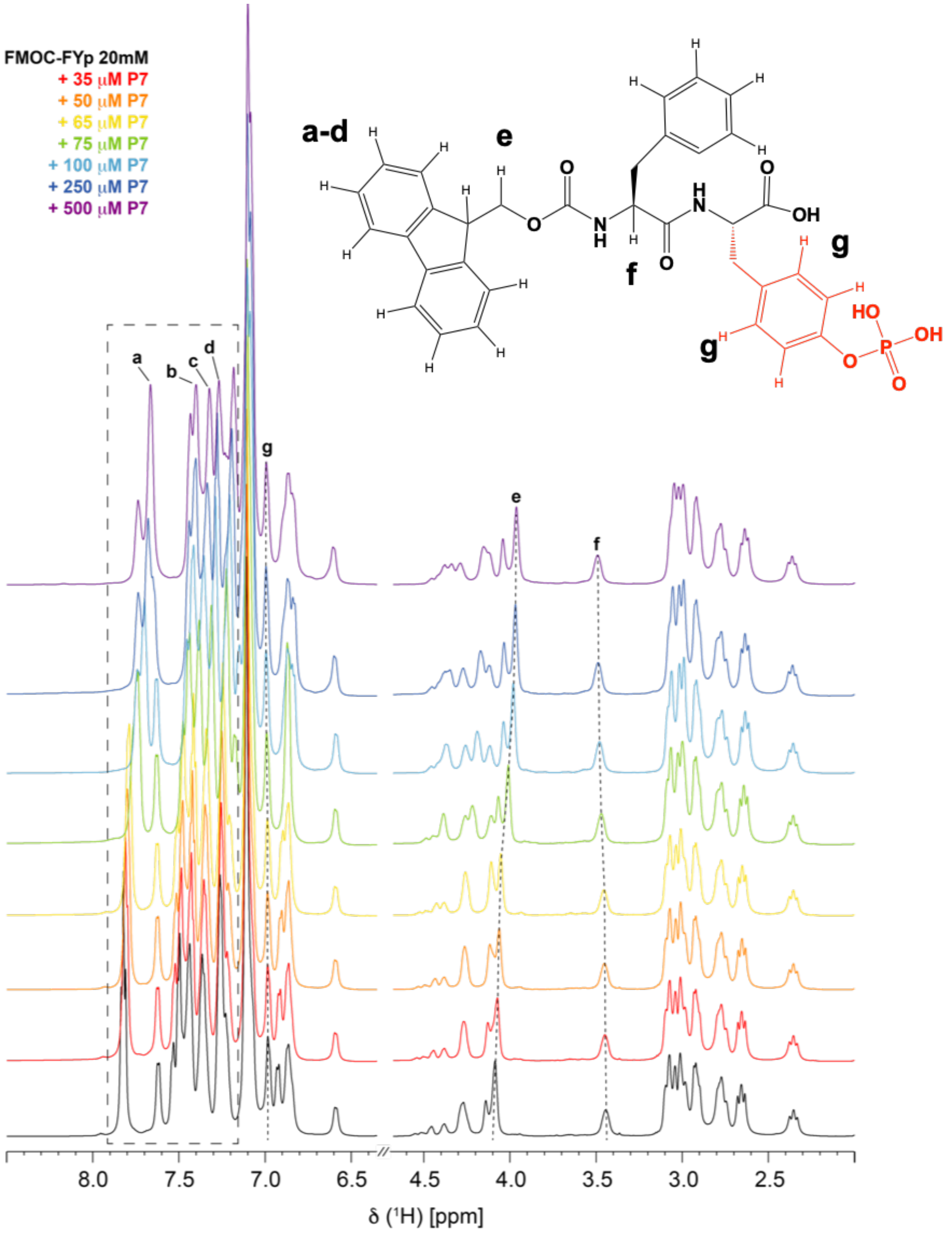

Supplementary Figure 5 – 1D-^{1}H NMR spectra of 20 mM Fmoc-F*p*Y titrated with increasing amounts of P7 in 100mM sodium phosphate pH 8 at 25 °C and 600 MHz.

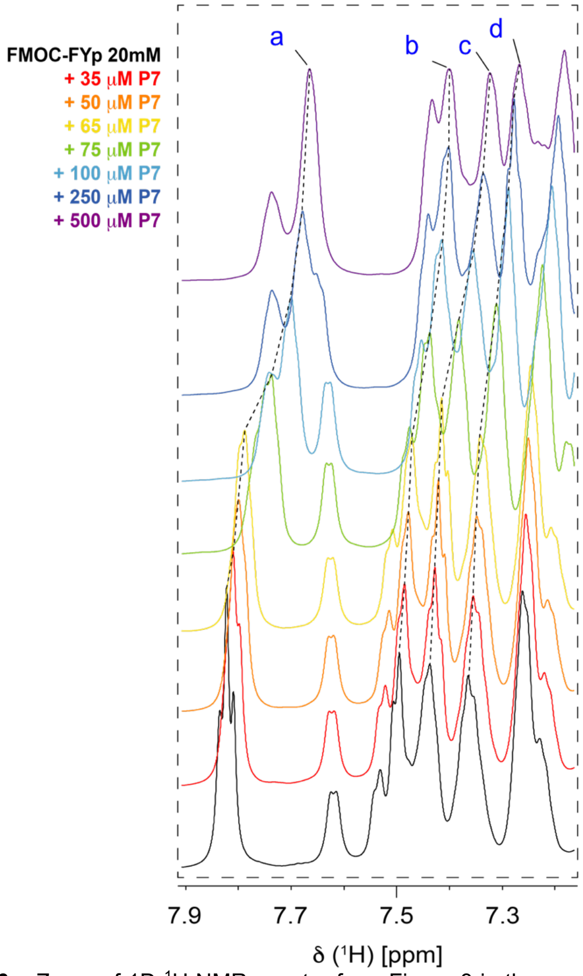

Supplementary Figure 6 – Zoom of 1D-^{1}H NMR spectra from Figure 3 in the range between 7.2-7.9 ppm of 20 mM Fmoc-F*p*Y titrated with increasing amounts of P7 in 100mM sodium phosphate pH 8 at 25 °C and 600 MHz.

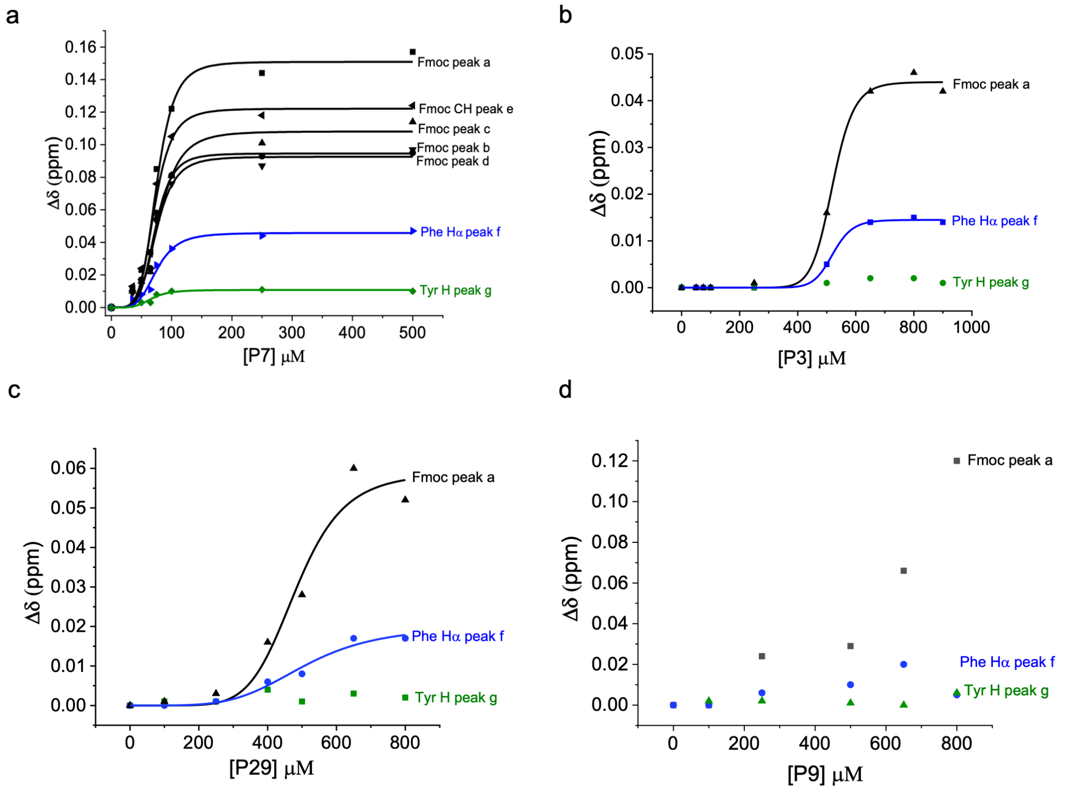

Supplementary Figure 7– Binding curve for 20 mM Fmoc-F*p*Y and (a) P7 (b) P3, (c)P29 and (d) P9 interaction obtained by NMR by monitoring the chemical shift changes of the Fmoc-F*p*Y proton signals identified in Fig.S4.

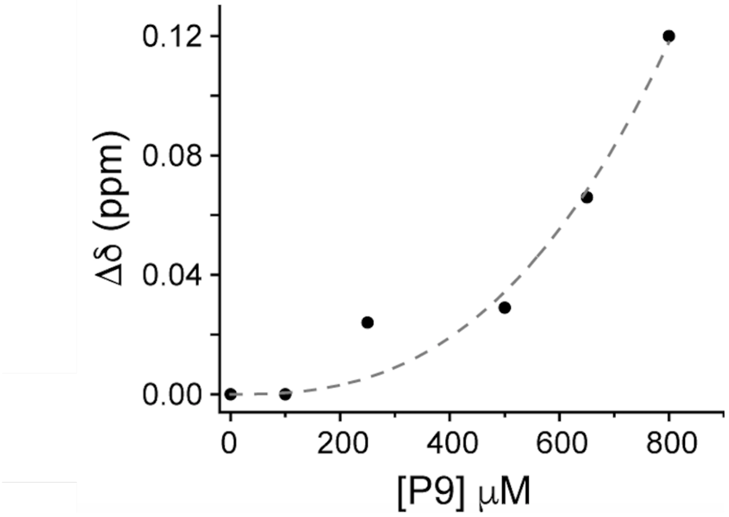

Supplementary Figure 8– 20 mM Fmoc-F*p*Y chemical shifts perturbation as a function of the P9 peptide concentration. The 1D ^{1}H NMR Chemical shift changes are of the most downfield Fmoc-F*p*Y aromatic proton signal.

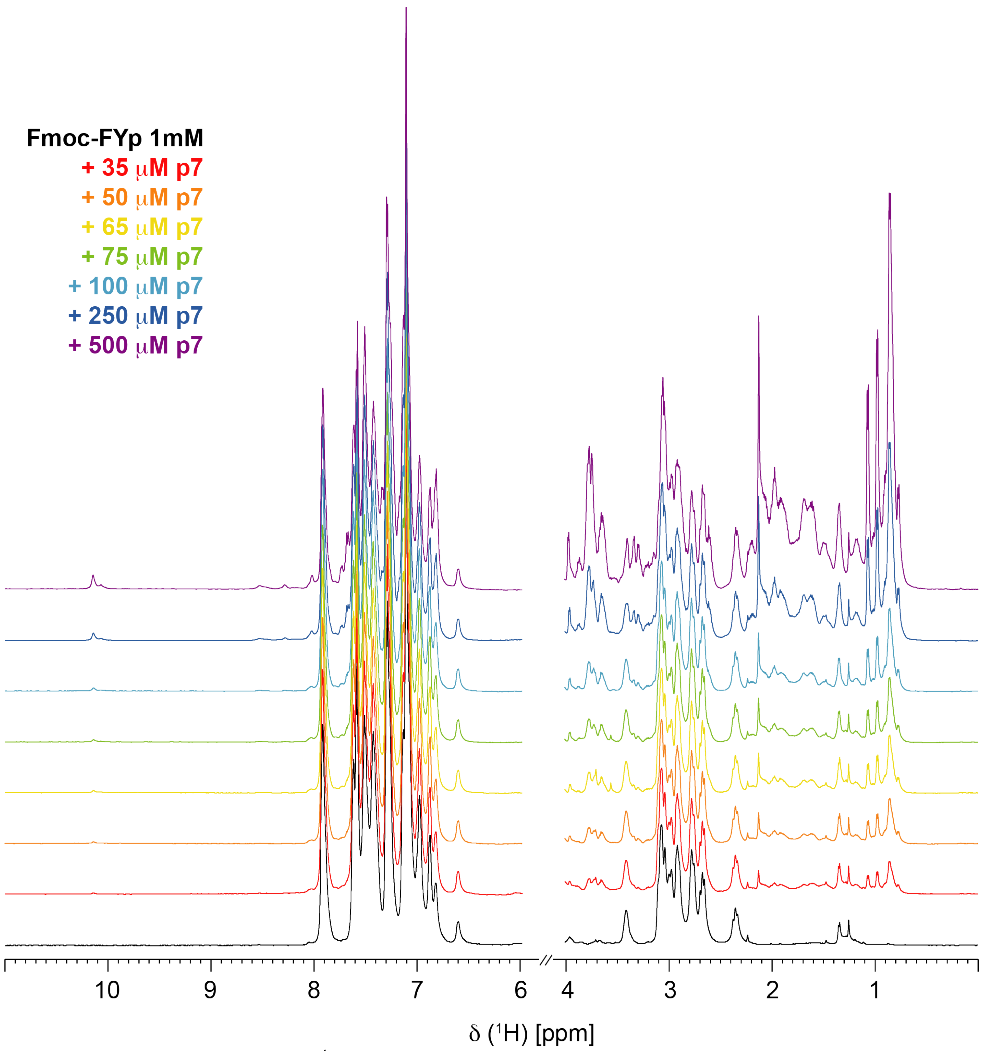

Supplementary Figure 9– 1D-^{1}H NMR spectra of 1 mM Fmoc-F*p*Y titrated with increasing concentrations of p7 in 100 mM sodium phosphate pH 8 at 25 °C and 600 MHz.

a

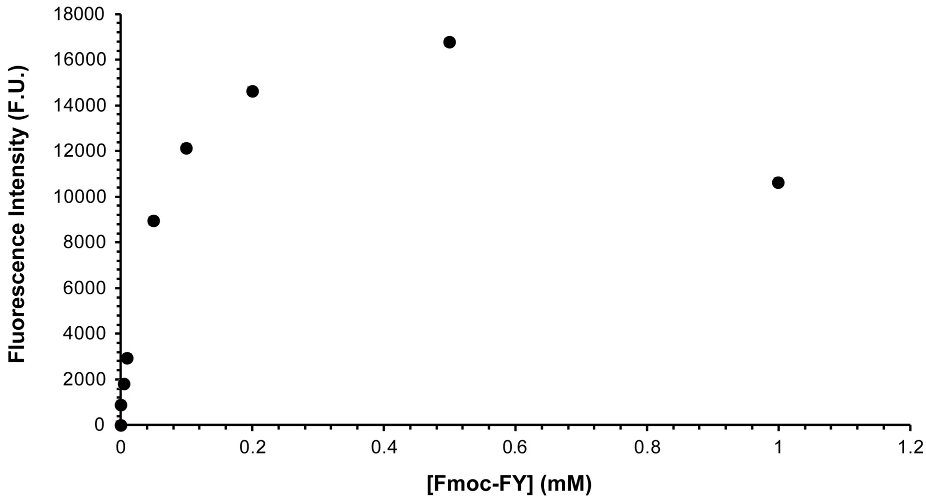

b

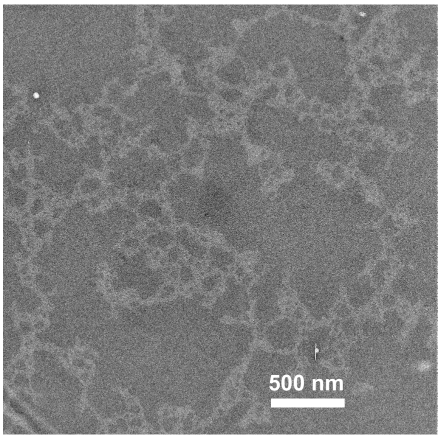

c

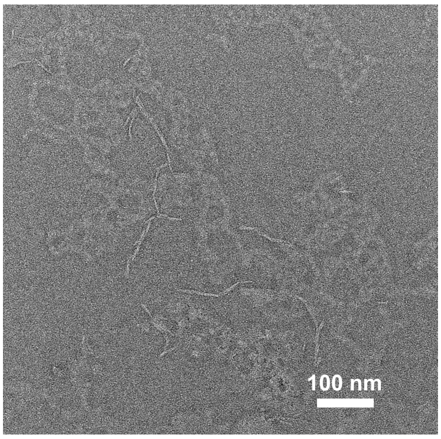

Supplementary Figure 10 – (a) Fluorescence emission spectra of Fmoc-FY at different concentrations, and representative TEM images of Fmoc-FY at **(b)** 0.1 mM and (**c**) 1 mM.

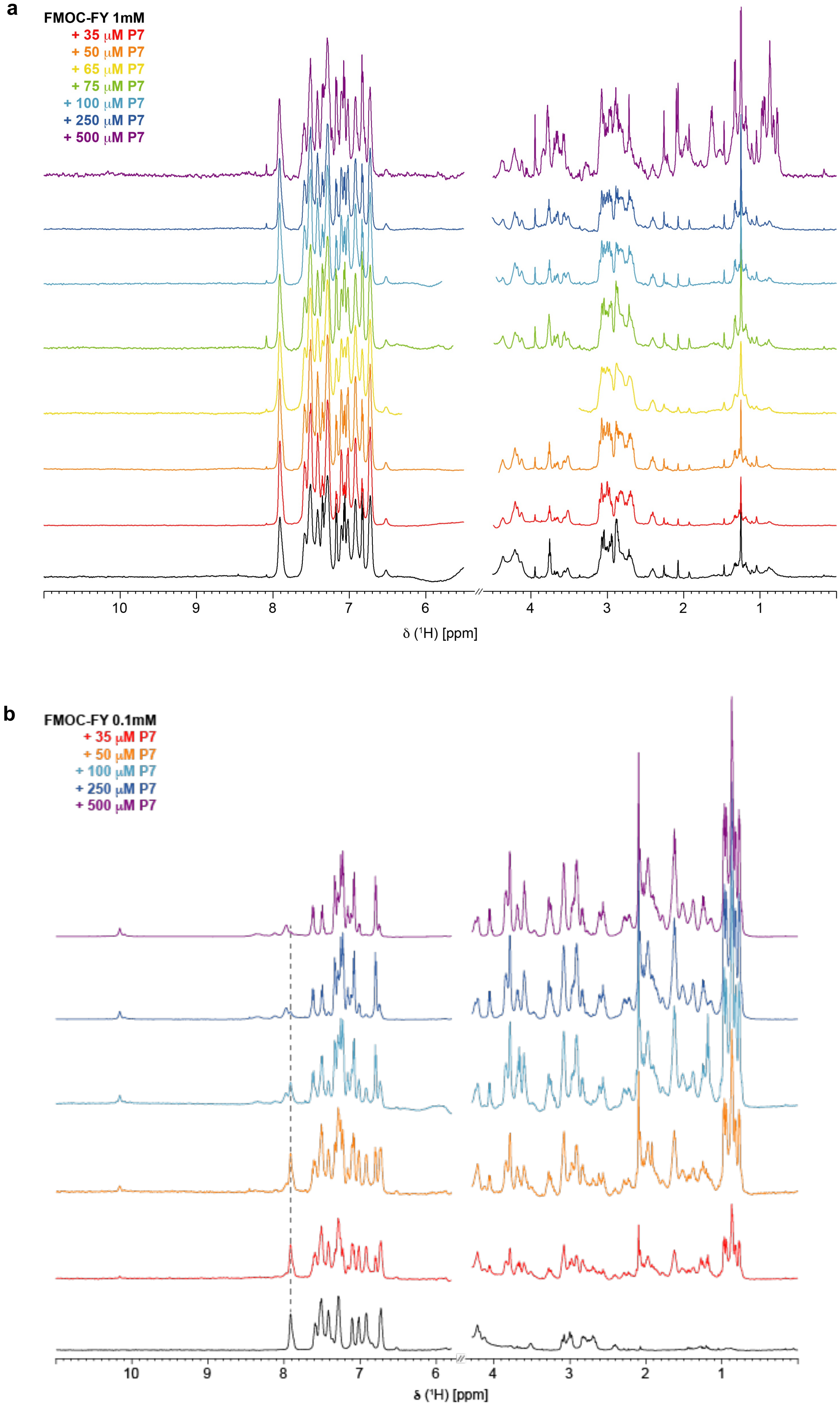

Supplementary Figure 11 1D-^{1}H NMR spectra of **(a)** 1 mM and **(b)** 0.1 mM Fmoc-FY titrated with increasing concentrations of P7 in 100 mM sodium phosphate pH 8 at 25 °C and 600 MHz. 1D ^{1}H NMR Chemical shift changes of the most downfield Fmoc-F*p*Y aromatic proton signal are shown.

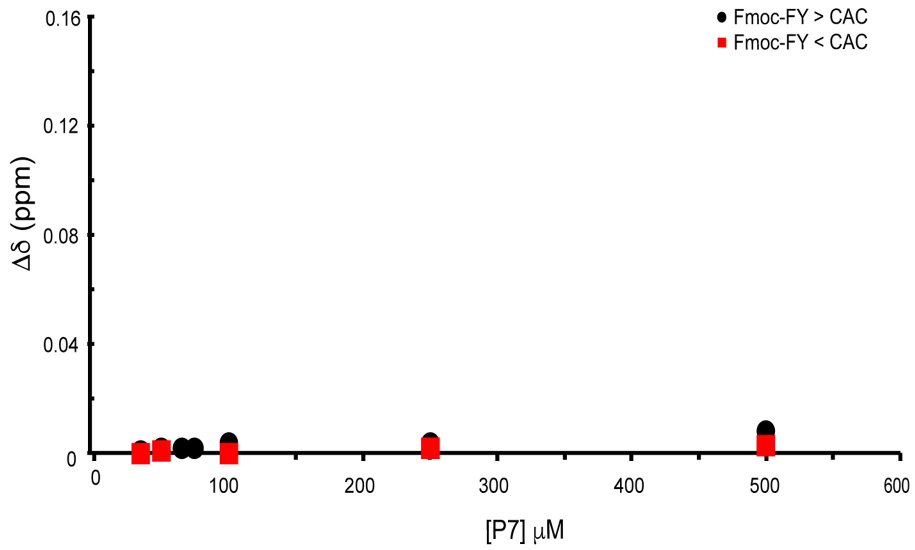

Supplementary Figure 12 Binding curve for 1 mM (above the critical assembly concentration - CAC) and 0.1 mM (below the CAC) Fmoc-FY and P7.

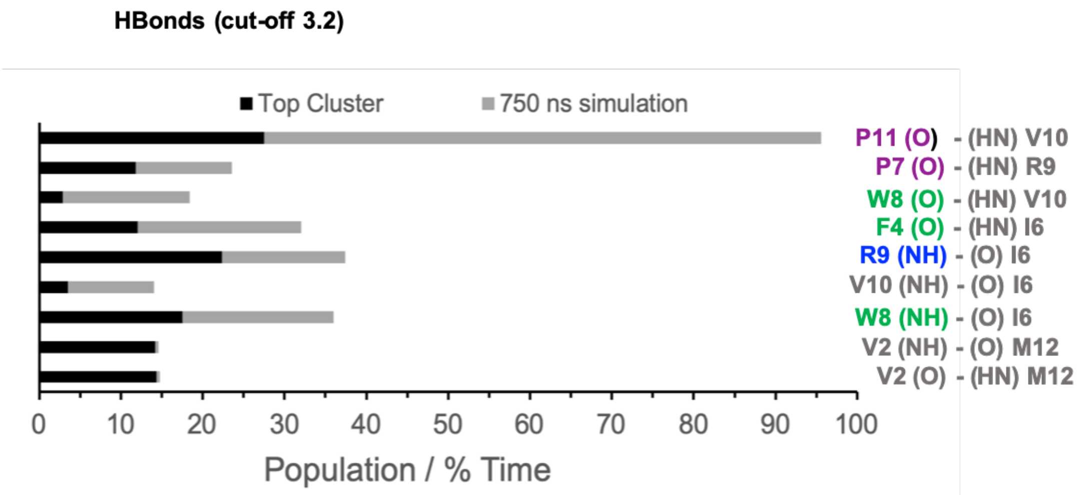

Supplementary Figure 13– Quantification of intramolecular H-Bond interactions observed during the computational simulations of the P7 peptide.

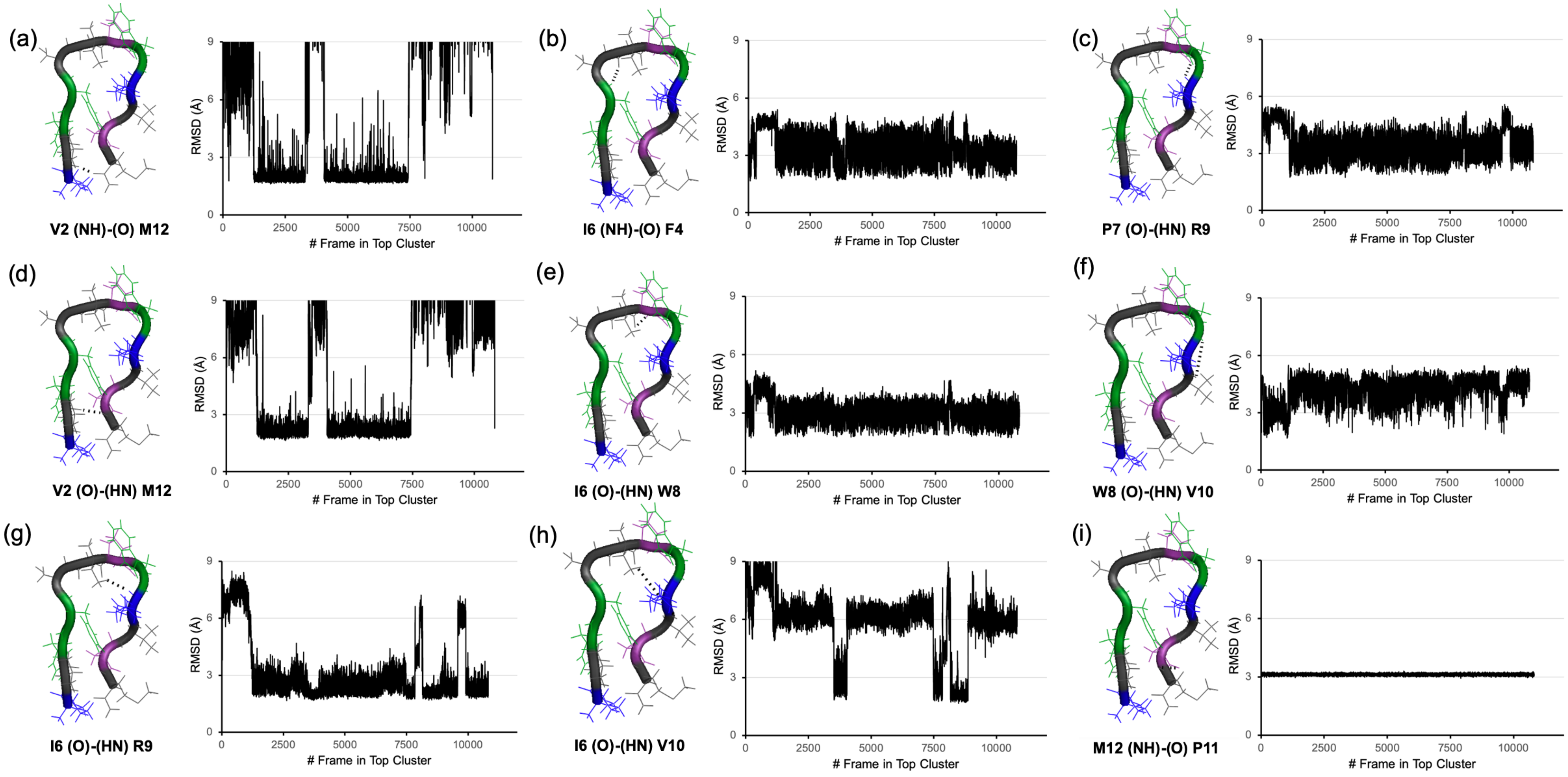

Supplementary Figure 14– Cooperative H-bonds interactions observed in the 30% of the total time of the computational simulation (750 ns). Dotted lines indicate the interaction that is analysed.

CH-π (cut-off 4.4)

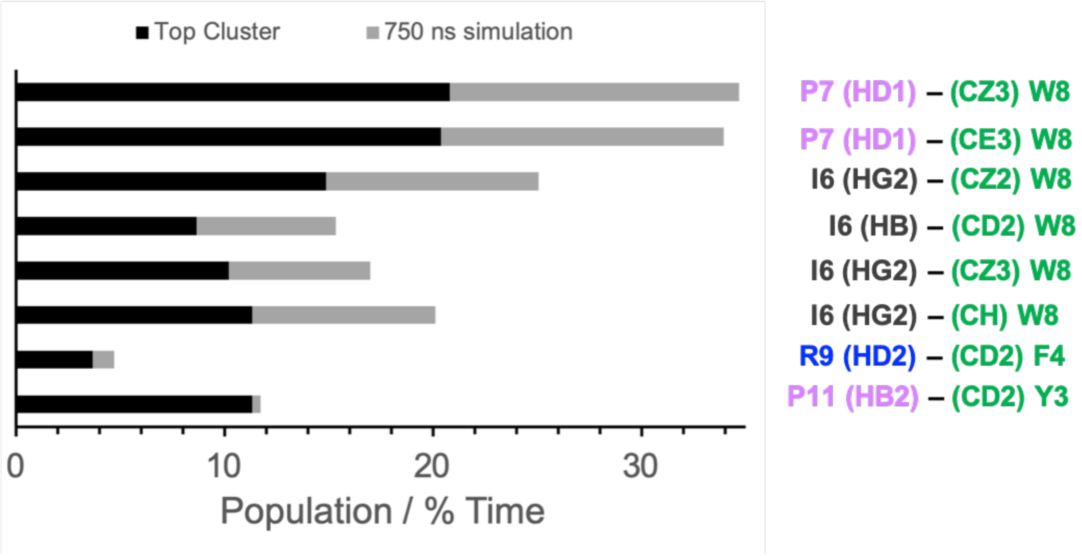

Supplementary Figure 15– Quantification of intramolecular CH-π interactions observed during the computational simulations of the P7 peptide.

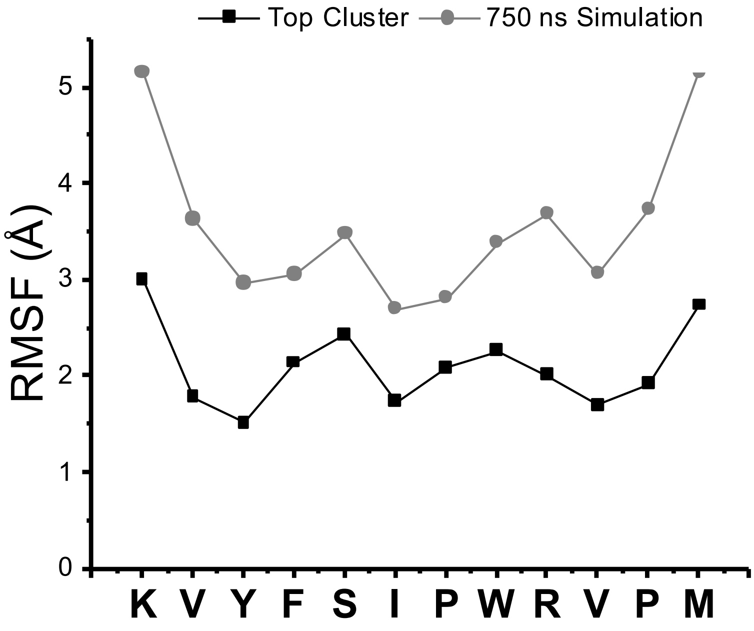

Supplementary Figure 16– Root mean square fluctuations (RMSF) of the P7 backbone atoms during MD simulation.

Alanine Mutants	
**P7**	KVYFSIPWRVPM-NH_2
**P7- K1A**	**A**VYFSIPWRVPM-NH_2
**P7- V2A**	K**A**YFSIPWRVPM-NH_2
**P7- Y3A**	KV**A**FSIPWRVPM-NH_2
**P7- F4A**	KVY**A**SIPWRVPM-NH_2
**P7- S5A**	KVYF**A**IPWRVPM-NH_2
**P7- I6A**	KVYFS**A**PWRVPM-NH_2
**P7- P7A**	KVYFSI**A**WRVPM-NH_2
**P7- W8A**	KVYFSIP**A**RVPM-NH_2
**P7- R9A**	KVYFSIPW**A**VPM-NH_2
**P7- V10A**	KVYFSIPWR**A**PM-NH_2
**P7- P11A**	KVYFSIPWRV**A**M-NH_2
**P7- M12A**	KVYFSIPWRVP**A**-NH_2

Supplementary Figure 17– Peptide Sequences of the alanine mutants.

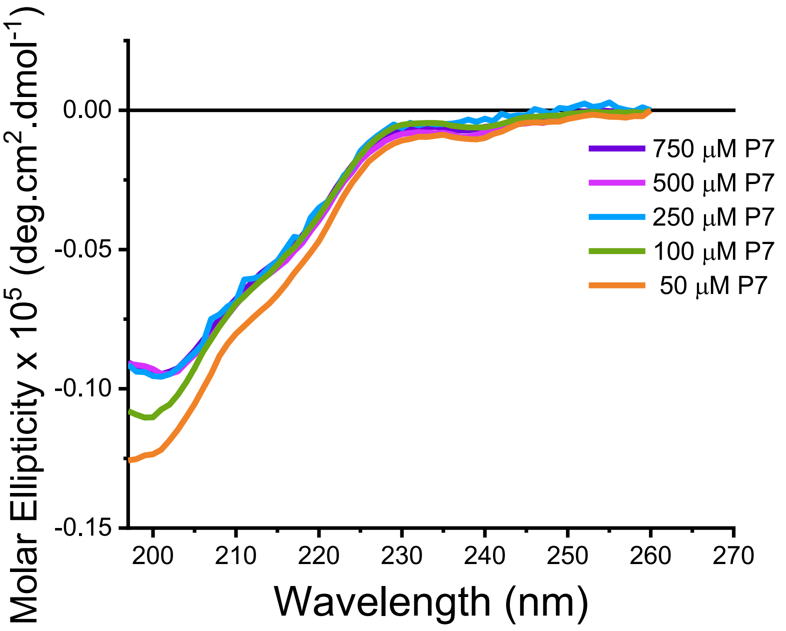

Supplementary Figure 18– CD spectra of P7 at different concentrations. Error bars represent standard deviations of at least three independent measurements

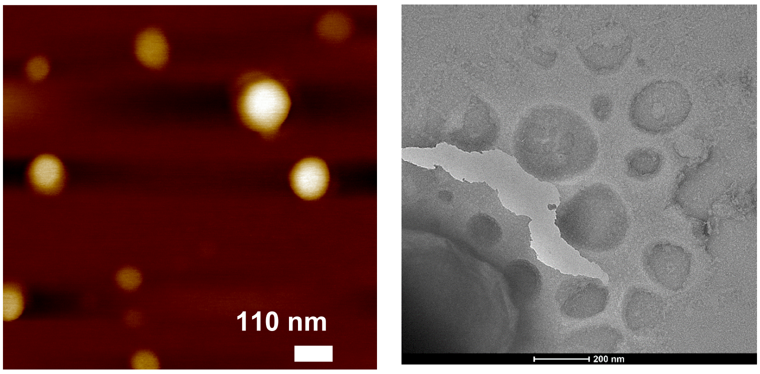

Supplementary Figure 19– AFM and TEM imaging at Critical Aggregation Concentration of P7 at 750 μM.

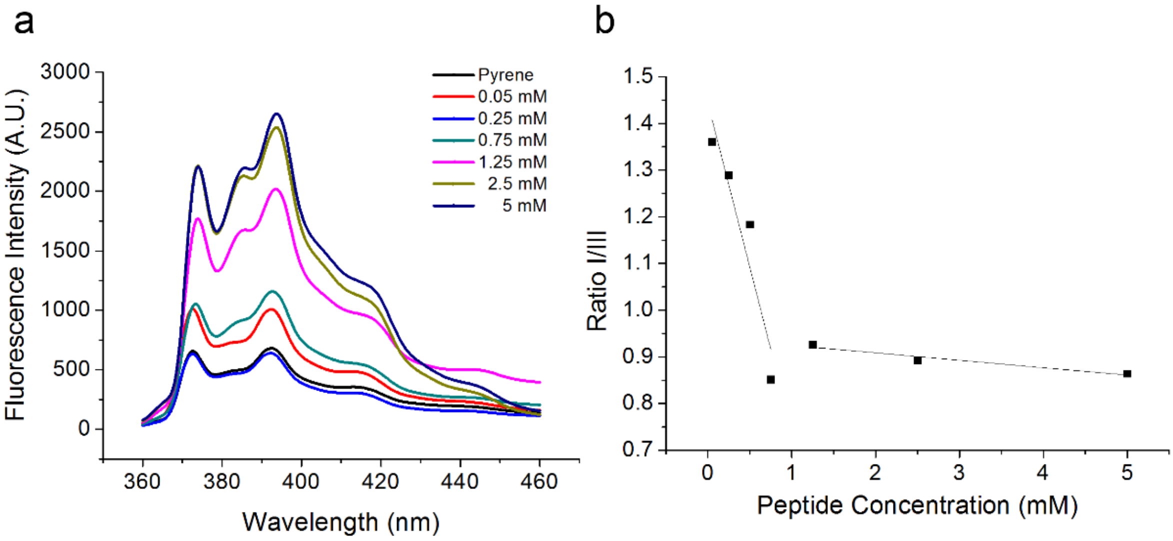

Supplementary Figure 20– Critical Aggregation Concentration. **(a**) Fluorescence emission spectra of pyrene at different peptide P7 concentrations, and **(b)** Plot of the ratio of I (λ_{max} = 372 nm) and III (λ_{max} = 384 nm) against peptide P7 concentration.

Discussion

P7 Self-assembly

The diffusion constant (D) of the supramolecular aggregates was described by the Strokes-Einstein equation[8]. The increase in P7 concentration resulted in slower diffusion, with a diffusion constant of 3 $\times 10^{-10}$ m^2 s^{-1} at 50 μM and a diffusion constant of 2 $\times 10^{-10}$ m^2 s^{-1} at 750 μM, suggesting the presence of larger supramolecular aggregates at higher concentrations of P7. P7 peptide tends to self-assemble into spherical aggregates, presenting a critical aggregation concentration of 750 µM (Fig. S15).

REFERENCES

1. M. J. Abraham, T. Murtola, R. Schulz, S. Páll, J. C. Smith, B. Hess and E. Lindahl, *SoftwareX*, 2015, **1-2**, 19-25.
2. K. Lindorff-Larsen, S. Piana, K. Palmo, P. Maragakis, J. L. Klepeis, R. O. Dror and D. E. Shaw, *Proteins* 2010, **78**, 1950-1958.
3. W. L. Jorgensen, J. Chandrasekhar, J. D. Madura, R. W. Impey and M. L. Klein, *J. Chem. Phys.*, 1983, **79**, 926-935.
4. U. Essmann, L. Perera, M. L. Berkowitz, T. Darden, H. Lee and L. G. Pedersen, *J. Chem. Phys.*, 1995, **103**, 8577-8593.
5. X. Daura, K. Gademann, B. Jaun, D. Seebach, W. F. van Gunsteren and A. E. Mark, *Angw. Chem.Int. Ed.*, 1999, **38**, 236-240.

6. J. W. Sadownik, J. Leckie and R. V. Ulijn, *Chem. Comm.*, 2011, **47**, 728-730.
7. G. Basu Ray, I. Chakraborty and S. P. Moulik, *J. Colloid Interface Sci.*, 2006, **294**, 248-254.
8. Y. Cohen, L. Avram and L. Frish, *Angew. Chem. Int. Ed.* 2005, **44**, 520-554.
